# Precision Microalgae: A New Conceptual Framework for Bioengineering Applications

**DOI:** 10.3390/bioengineering13091011

**Published:** 2026-08-31

**Authors:** Darissa Alves Dutra, Richard Luan Silva Machado, Mariany Costa Deprá, Adriane Terezinha Schneider, Eduarda Funari Machado, Mariane Bittencourt Fagundes, Leila Queiroz Zepka, Eduardo Jacob-Lopes

**Affiliations:** 1 Bioprocess Intensification Group, Federal University of Santa Maria, UFSM, Roraima Avenue 1000, Santa Maria 97105-900, RS, Brazil; darissa.alves@acad.ufsm.br (D.A.D.); richard.machado@acad.ufsm.br (R.L.S.M.); marianydepra@gmail.com (M.C.D.); adriane.schneider@acad.ufsm.br (A.T.S.); eduarda.funari@acad.ufsm.br (E.F.M.); zepkaleila@yahoo.com.br (L.Q.Z.); 2 CIIMAR-Interdisciplinary Centre of Marine and Environmental Research, University of Porto, Leixões Port Cruise Terminal, Avenida General Norton de Matos, 4450-208 Matosinhos, Porto, Portugal; mfagundes@ciimar.up.pt

**Keywords:** environmental heterogeneity, photobioreactor scale-up, physiological heterogeneity, digital twins, industrial biotechnology

## Abstract

Microalgae are promising platforms for biomass production, carbon capture, biofuels, and high-value bioproducts. However, despite significant advances in cultivation technologies, reactor engineering, and metabolic engineering, industrial implementation remains limited. This gap suggests that the main challenge of microalgae biotechnology lies not in the availability of productive strains or cultivation systems, but in managing the environmental and physiological heterogeneity that emerges during scale-up. This structured narrative review selected literature using predefined descriptors and relevance-based inclusion criteria and organized the evidence into five thematic domains encompassing cultivation-scale constraints, cellular physiology, bioengineering, precision technologies, and industrial translation. This review examines macrospatial bottlenecks related to light distribution, gas transfer, hydrodynamics, and reactor operation, alongside microspatial constraints involving cell cycle regulation, carbon allocation, metabolic adaptation, and stress responses. Recent advances in adaptive cultivation, real-time monitoring, artificial intelligence, digital twins, computational modeling, and bioengineering are discussed as tools to transform biological and environmental variability into actionable information. Based on concepts established in precision agriculture, this review proposes precision microalgae as a conceptual framework that integrates reactor engineering and cell physiology with three operational pillars: real-time monitoring, predictive modeling, and adaptive control. Its specific contribution is to connect currently fragmented technological and biological advances within a common framework for managing multiscale heterogeneity during cultivation and scale-up. Overall, the available evidence supports the operational logic of this framework, although its generalized effectiveness under industrial conditions remains to be demonstrated.

## 1. Introduction

Microalgae occupy a unique position in contemporary biotechnology. Few biological systems combine the capacity for photoautotrophic growth, remarkable metabolic versatility, and the ability to synthesize a wide range of commercially relevant compounds, including long-chain polyunsaturated fatty acids, high-value pigments, bioactive polysaccharides, and single-cell proteins. According to a commercial market research estimate, the global market for microalgae-derived products, which includes products based on *Spirulina*, *Chlorella* sp., *Dunaliella salina* and other microalgae in food and beverages, animal feed, cosmetics and related applications, was valued at approximately USD 15.51 billion in 2025, the base year of the report [1]. However, despite more than seven decades of cumulative research efforts in strain development, bioreactor engineering, and process optimization, industrial microalgae biotechnology remains confined to a relatively limited set of commercial applications, predominantly concentrated in high-value-added niche markets. This persistent gap between scientific promise and industrial reality is not incidental; it is structurally embedded in the very nature of the problem.

Previous reviews have identified a persistent divergence between the biological performance observed under controlled laboratory conditions and that achieved under pilot-, outdoor-, or industrial-scale cultivation [2,3]. For the purposes of this review, we use the term “yield gap” operationally as a metric-specific umbrella term for this scale-associated performance divergence, rather than as a single universal variable. Depending on the original study, the gap may be characterized using volumetric productivity (g L^−1^ d^−1^), areal productivity (g m^−2^ d^−1^), photosynthetic yield or efficiency (%), target-product concentration (e.g., mg L^−1^), or cellular product content (e.g., mg g^−1^ dry biomass). The specific indicator, unit, reference condition, and validation scale are identified in each case. Because these metrics represent distinct dimensions of performance, values based on different indicators are not treated as interchangeable or quantitatively pooled.

The dominant explanation for this divergence has focused on biological limitations, insufficient photosynthetic efficiency, inadequate metabolic flux towards target products, or susceptibility to specific environmental stresses. The weight of recent evidence suggests that this approach incorrectly identifies the main source of the problem. The fundamental limitation of contemporary microalgae production is not, in most cases, the intrinsic metabolic capacity of the cultured cells. The difficulty lies in the current system’s ability to manage the dynamic and multi-scale environmental and physiological heterogeneity inherent in large-scale cultivation and to translate this heterogeneity into actionable operational information [4,5,6].

In laboratory photobioreactors (PBRs), cells are exposed to relatively homogeneous, rigorously controlled conditions of irradiance, temperature, dissolved-gas concentrations, and nutrient availability. On an industrial scale, this homogeneity ceases to exist [7,8]. Spatial and temporal gradients in the distribution of light, dissolved oxygen, carbon dioxide (CO_2_) availability, pH, and hydrodynamic shear become defining characteristics of the cultivation environment, not disturbances to be corrected, but permanent conditions to be managed [9]. Individual cells within the same culture follow substantially different microenvironmental trajectories over time, experiencing irradiance sequences, nutrient availability, and mechanical stress that diverge markedly from the population-average conditions recorded by process sensors [6,10]. Consequently, identical cultures can produce profoundly different results, depending on which cells physiologically predominate at a given time, a phenomenon that control strategies based on averages are structurally incapable of capturing. The transition from laboratory to industrial scale is, in this sense, not only a change in volume but also a fundamental shift in the biological problem.

Historically, this challenge has been approached from at least three distinct perspectives, each of which has yielded genuine, albeit ultimately partial, advances. Photobioreactor engineering has sought to reduce physical gradients by optimizing reactor geometry, mixing, and gas transfer. Metabolic engineering and synthetic biology have expanded the cellular capacity to direct carbon toward products of interest and increase tolerance to environmental stresses. In parallel, process monitoring technologies have increased the ability to track the state of cultivation in real-time domains [11,12,13,14]. The main limitation of these approaches is, considered individually, not the absence of results; the literature documents a wide range of quantitatively significant improvements in all of them. The limitation is that advances in one dimension often introduce new constraints in another.

A conceptual parallel can be drawn with precision agriculture. For much of the 20th century, agronomic management relied on uniform prescriptions derived from average values, implicitly assuming that large, cultivated areas responded uniformly to the same inputs. The development of precision agriculture arose from the recognition that this premise was inadequate and that spatial heterogeneity should not be treated merely as a source of variability but as a source of information for management [15,16]. The incorporation of sensors, monitoring systems, and predictive models has made it possible to replace uniform strategies with adaptive and data-driven interventions [17,18]. Fundamentally, precision agriculture did not result from a new biological discovery about crops, but from a new way of interpreting the relationship between biological variability and decision-making. We argue that a conceptually similar transformation is needed for microalgae biotechnology.

Several lines of research are already advancing, albeit in a fragmented way, in this direction. The concept of precision fermentation has driven the development of more controlled and data-driven processes [19,20,21,22], while adaptive cultivation approaches have demonstrated the ability to dynamically adjust operating conditions in response to the physiological state of the culture [14,23].

In parallel, the integration between machine learning, process sensing, and real-time monitoring has expanded the ability to estimate biomass and infer physiological states in complex cropping systems [14,24]. Digital twin structures have been proposed to integrate hydrodynamic, physiological, and metabolic models, allowing for prospective simulation of crop dynamics under varying conditions [25,26]. At the same time, the development of lineages supported by multiomics data, computational metabolic modeling, and synthetic biology is approaching systems-oriented biological engineering approaches, in which genetic interventions are designed considering integrated metabolic and regulatory networks, rather than isolated genes or pathways [11].

What the current literature still lacks are not technologies or evidence of feasibility, but a conceptual framework capable of integrating these scattered advances into a common logic. Although terms such as “smart farming,” “smart bioprocessing,” or “data-driven algae production” appear with increasing frequency [27,28,29], they typically describe specific technological applications, rather than a conceptual architecture capable of simultaneously articulating the biological and engineering scales involved in microalgae production. In this context, we propose the concept of *precision microalgae* as an integrative paradigm for the next generation of microalgal biotechnology. Instead of treating physiological, environmental, and operational variability as a source of uncertainty to be minimized, this approach recognizes it as an inherent element of cultivation and an opportunity to guide management decisions, control strategies, and strain development.

The goal is not to impose homogeneous conditions on an inherently heterogeneous biology, but to incorporate the information generated by this heterogeneity into the decision-making process throughout cultivation. To support this proposal, this article examines the macrospatial bottlenecks associated with culture systems and the microspatial bottlenecks that emerge at the cellular level, critically evaluating current bioengineering strategies and the recurring trade-offs between growth, physiological stability, and product accumulation. Based on this diagnosis, the main technological components that underpin the *precision microalgae* paradigm are discussed, as well as the translation barriers that still limit its industrial implementation. This paper does not propose a specific technology or a completed research program, but rather a conceptual framework aimed at integrating currently dispersed advances towards more predictive, adaptive, and biologically informed production systems.

## 2. Methodological Approach

To critically evaluate the scientific and technological advances associated with scaling up microalgae production, this study was conducted through a structured literature review, supported by a qualitative analysis of the main challenges and innovations reported for large-scale microalgae cultivation [30,31].

The review was designed to explore the interconnectedness of culture systems, cell physiology, bioengineering strategies, and emerging digital technologies, rather than focusing on a single biological or engineering aspect. The literature search was initially conducted using broad descriptors related to the cultivation, scale-up, and bioengineering of microalgae, including “microalgae cultivation,” “scale-up of photobioreactors,” “microalgae bioengineering,” and “microalgae physiology.” Additional descriptors were progressively incorporated to encompass emerging concepts associated with intelligent bioprocesses and adaptive cultivation systems, including “bottlenecks in microalgae production”, “digital twins”, “artificial intelligence in biotechnology”, “CRISPR microalgae”, “advances in microalgae bioengineering”, and “adaptive process control”.

To improve analytical coherence, the retrieved publications were organized into five thematic areas that correspond to the conceptual framework proposed in this review: (i) macrospatial bottlenecks associated with cultivation and scale-up systems, including light heterogeneity, gas transfer limitations, hydrodynamics, and reactor design; (ii) microspatial bottlenecks associated with cell physiology, including cell cycle regulation, nutrient sensing, carbon assimilation, oxidative stress, and phenotypic heterogeneity; (iii) cell bioengineering strategies aimed at improving mechanical robustness, stress tolerance, and metabolic performance; (iv) precision bioengineering approaches integrating real-time monitoring, computational biology, digital twins, artificial intelligence, and synthetic biology; and (v) translational bottlenecks affecting the industrial implementation of advanced microalgae technologies, including reproducibility, genetic stability, regulatory constraints, and scalability.

**Figure 1:**
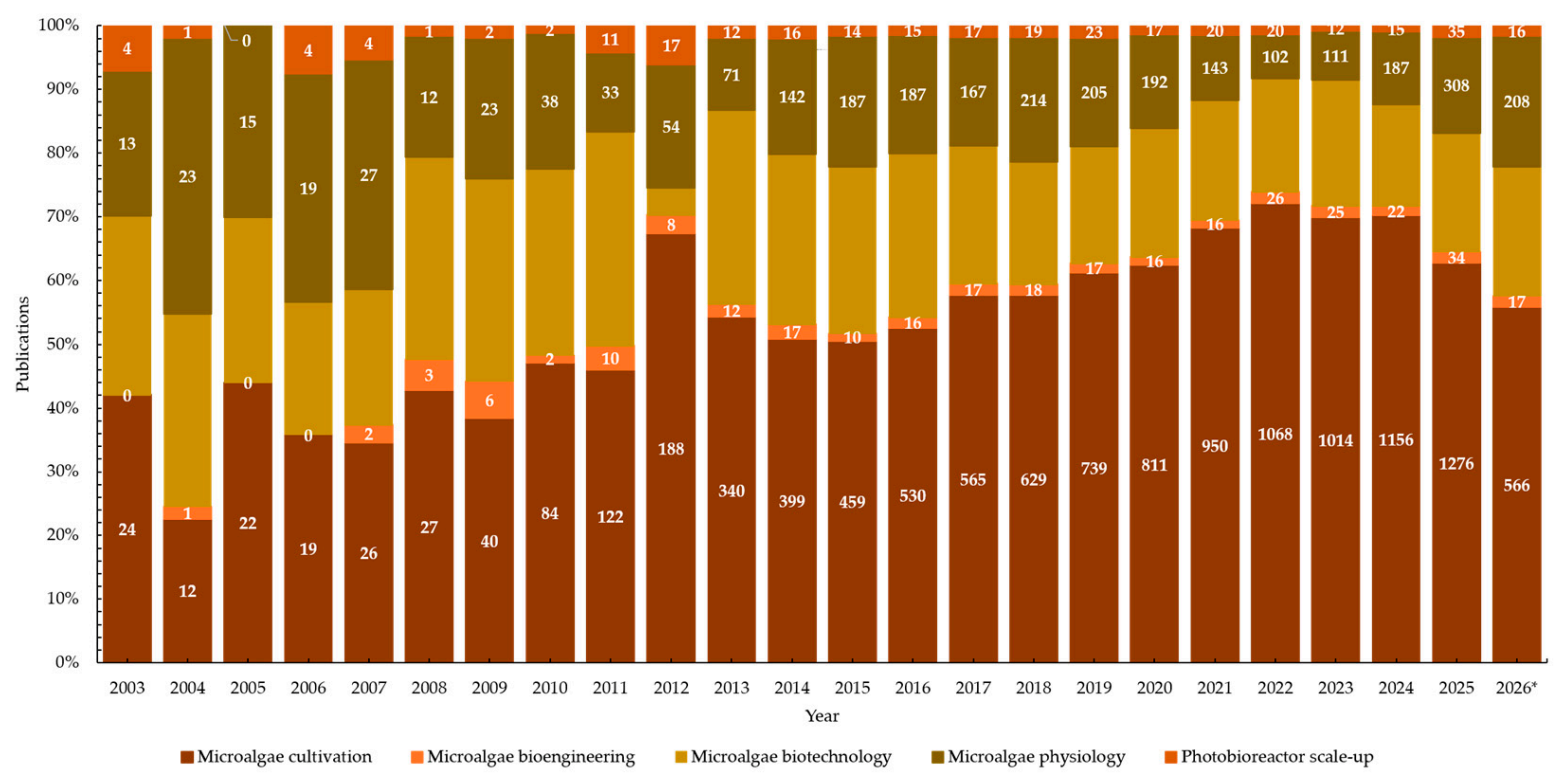
Evolution of the scientific literature related to microalgae cultivation, physiology, bioengineering, and scale-up from 2003 to 2026 (* indicates until June 2026).

Given the multidisciplinary nature of the topic, peer-reviewed research articles, review articles, book chapters, technical reports, and articles with an industrial perspective were included. The search prioritized publications indexed in the main scientific databases and publisher platforms, with an emphasis on studies published between 2003 and 2026, a period that encompasses the fundamental biological and engineering contributions to microalgae cultivation, the consolidation of research in scale-up, and the progressive convergence between reactor engineering, computational modeling, and artificial intelligence in the optimization of bioprocesses (Figure 1).

The inclusion criteria comprised studies that: (i) investigated microalgae cultivation systems and scaling-up challenges; (ii) evaluated physiological, metabolic, or cellular responses to environmental fluctuations; (iii) explored metabolic engineering, synthetic biology, or strain improvement strategies; (iv) addressed process monitoring, automation, computational modeling, digital twins, or artificial intelligence applications; and (v) discussed industrial implementation, technological readiness, or translational aspects of microalgae biotechnology. Duplicate records, studies unrelated to microalgae, publications lacking experimental or conceptual relevance for scale-up and cell engineering, and studies with insufficient methodological information were excluded.

For analytical purposes, the selected studies were classified according to two complementary dimensions. First, the publications were grouped by biological scale, encompassing phenomena at the bioreactor scale, population-level responses, and cellular-level mechanisms. According to the studies, they were categorized according to the nature of the bottleneck investigated, including engineering constraints, physiological limitations, bioengineering interventions, and precision biotechnology solutions. This integrative framework enabled the identification of critical interactions, trade-offs, and knowledge gaps that currently limit the development of predictive, scalable, and biologically informed microalgae production systems, ultimately supporting the conceptual proposal of *precision microalgae* as a next-generation paradigm for industrial microalgae biotechnology.

## 3. Macrospatial Bottlenecks: Why Do Large-Scale Systems Behave Differently?

Microalgae biotechnology holds well-documented productive promise; under optimized laboratory conditions, microalgae exhibit growth rates, photosynthetic efficiencies, and product accumulations that far surpass conventional terrestrial cultures. The problem is that this promise was built in fractions of a liter. When the system scales to cubic meters, the physical environment ceases to be controllable in the same way, and performance collapses in ways that models based on laboratory averages cannot predict. The main evidence for this collapse is not in volume, but in the fact that, on a large scale, each cell begins to experience distinct environmental trajectories of light, dissolved gases, temperature, and shear, creating heterogeneous physiological microenvironments within the same reactor [4]. The relevant question, therefore, is not how much the cell can produce in the laboratory, but under what physical conditions it is able to sustain that production as the system grows.

A comparison between different geometries clearly illustrates this tension. Closed photobioreactors (flat panel PBR) show an annual productivity of 10.4–35.6 g m^−2^ d^−1^ in outdoor cultivation, compared to 5.5–19.7 g m^−2^ d^−1^ in open raceway systems [32], suggesting an apparent productivity advantage of up to approximately twofold. However, this advantage is not an intrinsic consequence of reactor closure; it results from the greater control of optical path, circulation, gas exchange, and temperature that closed configurations attempt to maintain. In an outdoor comparison using the same cultivation area, reducing culture depth from 20 to 1.1 cm increased mean in-culture irradiance by approximately 45%, and raised areal biomass productivity from 11.46 to 31.92 g m^−2^ d^−1^ [33]. The gain was therefore achieved by shortening the light path and reducing the culture volume from 2200 to 280 L, rather than by demonstrating that the same optical advantage could be preserved as reactor dimensions increased. Thus, the conditions that improve light utilization may simultaneously restrict the amount of culture accommodated per unit area and require a substantially different hydrodynamic configuration. Controlled experiments reinforce the optical basis of this trade-off. Under simulated solar illumination, a V-shaped *Chlorella vulgaris* cultivation system increased areal biomass productivity from 20.7 to 52.0 g m^−2^ d^−1^ and photosynthetic efficiency from 1.40% to 3.52% compared with a flat configuration [34]. However, these results were obtained in laboratory reactors of 128–684 mL and therefore demonstrate the potential of optical redistribution rather than its preservation at production scale.

The opposite design strategy, accommodating more culture by increasing reactor depth, introduces a different limitation because nominal volume does not necessarily correspond to effectively mixed and productive volume. In a 4.5-m^3^ pilot-scale high-depth raceway pond, regions with local velocities below approximately 0.1 m s^−1^ were classified as dead or stagnant zones. Flow deflectors reduced these regions by 28% and decreased the pressure drop across the 180° bends by 49%, with a length-to-width ratio of approximately 10 providing the most favorable configuration [35]. The importance of this result lies not only in the effectiveness of the deflectors, but in the underlying failure they reveal: bulk-average operating conditions can conceal poorly renewed regions within the same reactor. Moreover, because the intervention was optimized for a specific geometry, it cannot be treated as a transferable solution for the pH, gas, and nutrient heterogeneity generated in reactors with different dimensions and circulation regimes.

Recovering environmental uniformity through more intensive circulation, however, introduces hydrodynamic and energetic penalties. Across multi-tubular airlift, helical-tubular, and flat-panel reactors, biomass productivity did not differ significantly under nutrient-replete conditions, whereas specific power requirements ranged from 0.79 to 6.8 W L^−1^. This discrepancy shows that greater engineering intensity can increase the energetic cost of maintaining the culture without producing a corresponding biological gain. The same dissociation became more pronounced during outdoor cultivation of *Limnospira platensis*; a 2.6-m^3^ tubular PBR achieved approximately threefold higher volumetric productivity than a 3.6-m^3^ raceway but required 102.4 rather than 14–17 MJ kg^−1^ biomass. At industrial scale, the energy requirement of the tubular system reached 352.6 MJ kg^−1^ biomass, approximately 50-fold that of industrial raceways, despite yielding less than a twofold productivity advantage [36]. These results demonstrate that recovering physical control at larger scales does not eliminate the scale-up bottleneck; it transfers part of the limitation from biomass formation to energy demand and cellular integrity.

Geometry also influences gas management by determining how efficiently circulation promotes gas–liquid mass transfer and, consequently, the removal of photosynthetically generated O_2_ and the availability of supplied CO_2_. A thin-layer photobioreactor operating at a culture depth of 1 cm achieved a mixing time of 13.6 s and an O_2_ volumetric mass-transfer coefficient (kLa) of approximately 143 h^−1^ [37]. Although this result illustrates the mass-transfer advantage provided by a short liquid path and rapid circulation, it does not demonstrate that the same performance can be maintained when culture depth or reactor volume increases. Geometry therefore does not remove the dominant constraints of cultivation; it redistributes them among light utilization, gas–liquid mass transfer, hydrodynamic stress, energy demand, and biomass productivity. The relevant engineering question is consequently not which configuration maximizes a single performance metric, but which provides the most favorable balance among these constraints at the intended scale and operating conditions.

Seasonal variability reinforces this diagnosis. In industrial raceway cultivation of *Limnospira platensis* on a scale of 1000–4000 m^2^, the average annual productivity ranged from 5.1 to 5.6 g m^−2^ d^−1^, with peaks of 7.5–7.6 g m^−2^ d^−1^ between April and September and a drop of more than 60% in the winter months [38]. A comparable seasonal dependence was observed during 16 months of *Nannochloropsis oculata* cultivation in a 2.5-m^3^ outdoor pilot-scale raceway, where average areal productivity increased from 0.72–1.27 g m^−2^ d^−1^ in winter to 3.62 g m^−2^ d^−1^ in summer [39]. Together, these results demonstrate that pronounced seasonal productivity losses occur across different species and cultivation scales, although their magnitude remains dependent on the strain, geographic location and local operating conditions. This pattern reveals a bottleneck that no reactor geometry optimization can fully resolve: industrial productivity is not determined by the maximum the system can achieve under favorable conditions, but by the output that can be sustained throughout the year. Models incorporating local irradiance, temperature, and photoperiod similarly predicted geographic differences of 21–46% in biomass productivity and 43–63% in lipid productivity even within the same climate type. Extending this analysis across subtropical and mid-latitude regions, annual average biomass productivity decreased from 0.144 to 0.049 g L^−1^ d^−1^, while annual average lipid productivity decreased from 0.015 to 0.005 g L^−1^ d^−1^, corresponding to an approximately threefold difference between climatic zones [40]. Therefore, these results demonstrate why geographic location, and seasonal climate cannot be treated as secondary operating variables. More importantly, they show that extrapolating production potential from short cultivation periods or peak yields systematically overestimates year-round industrial performance.

Temperature represents an additional source of heterogeneity that is frequently obscured in scale-up analyses. Closed reactors provide greater physical isolation from the external environment, but their large, irradiated surfaces can accumulate thermal loads rather than ensure uniform temperature control. During outdoor cultivation of *Nannochloropsis* sp., 140-L closed flat-panel PBRs reached 31.0–33.8 °C, compared with 25.8 °C in a 200-L open raceway, while an unprotected closed reactor reached 43 °C and underwent a rapid decline in cell density [41]. The limitation is not restricted to whole-reactor heating. Modeling of an outdoor horizontal-loop tubular PBR containing *Chlorella vulgaris* predicted temperatures ranging from 24.85 to 38.45 °C within the same reactor, with the highest values occurring after the U-loop [42]. Thus, a single bulk-temperature measurement can conceal local thermal trajectories that exceed the physiological range experienced elsewhere in the culture. Because temperature affects metabolic kinetics, photosynthetic efficiency, and carbon assimilation [43], closed-reactor control cannot be inferred from enclosure alone; it depends on whether heat can be removed at the rate and spatial resolution required as reactor dimensions increase. The temperature threshold is also strongly strain-dependent. In controlled flat-panel PBRs, thermotolerant *Picochlorum* sp. BPE23 exhibited its highest daily dilution rate under a diel cycle peaking at 40 °C. However, the rate decreased from 1.22 ± 0.06 d^−1^ at the 40 °C peak to 0.61 ± 0.03 and 0.29 ± 0.02 d^−1^ at peaks of 45 and 47.5 °C, respectively [44]. Thus, some strains can remain productive at 40 °C and survive brief exposure to still higher temperatures, but this tolerance cannot be generalized across species or assumed to ensure year-round outdoor performance.

The productive consequences of thermal variability become evident only when performance is evaluated over an annual rather than an instantaneous timescale. Simulations of outdoor *Dunaliella salina* cultivation across five climatic conditions predicted 76 and 131 days of heat-induced culture collapse per year under Mediterranean and arid climates, respectively. When a five-day reinoculation period was included after each collapse, annual productivity decreased by approximately 35% and 40% [45]. Open systems exchange part of this daytime overheating risk for greater exposure to ambient and nocturnal temperature fluctuations, particularly in temperate and higher-latitude regions [38]. Thus, neither enclosure nor evaporative cooling eliminates thermal limitation; each configuration produces a different temporal pattern of thermal stress. Because these losses are poorly represented by measurements collected during favorable cultivation periods, neglecting collapse, recovery, and seasonal downtime leads to systematically optimistic estimates of industrial performance.

Within each system, the dynamics of light represent the first mechanism by which the productive promise is fragmented. As cell density increases, self-shading reduces light penetration, and the cells begin to circulate between illuminated regions and dark zones in cycles determined by the hydrodynamics of the reactor. As biomass concentration increases, self-shading restricts light penetration, while hydrodynamic circulation repeatedly transfers cells between illuminated and dark regions.

In outdoor pilot-scale flat-panel membrane PBRs dominated by *Chlorella*, reducing the light path from 25 to 10 cm increased volumetric biomass productivity from 66 ± 6 to 194 ± 24 mg VSS L^−1^ d^−1^ and areal biomass productivity from 15.7 ± 1.4 to 20.0 ± 2.4 g VSS m^−2^ d^−1^. The corresponding photosynthetic efficiency also increased from 3.02 ± 0.36% to 5.40 ± 1.63% [46]. However, the higher biomass concentration achieved in the narrower reactor preserved substantial self-shading, showing that shortening the optical path alleviated but did not remove the interaction between biomass accumulation and light limitation. Moreover, when the path is reduced to only 2–8 mm, the opposite limitation may emerge: the absence of dark zones, which in conventional systems function as photochemical recovery windows, favors early photoinhibition even under moderate irradiances [13]. Thus, reducing the optical path can alleviate self-shading, but excessively short light paths may shift the system from light limitation toward excessive light exposure. The flashing-light effect, often presented as a solution to this problem, exemplifies a recurring pattern in microalgal biotechnology: strategies that demonstrate promising results under controlled laboratory conditions do not always maintain performance during scaling up.

The flashing-light effect illustrates the same problem of context dependence. Although pulsed illumination increased *Nitzschia* sp. biomass by 137.6% in a membrane PBR [47], this gain was obtained under experimental conditions that did not reproduce the combination of high cell density, long transport distances, and heterogeneous optical trajectories characteristic of industrial cultivation. The result demonstrates the biological potential of intermittent illumination, but not its preservation during scale-up. At production-relevant densities, self-shading may persist even after the optical path is shortened, while the mixing intensity required to impose favorable light–dark cycles introduces additional energy and shear constraints. The central limitation is therefore not whether flashing light can stimulate photosynthesis under controlled conditions, but whether the required exposure frequency and distribution can be delivered uniformly and energetically at large scale.

Gas limitation operates on analogous logic and frequently overlaps with light limitation. Phototrophic reactors must simultaneously supply carbon dioxide (CO_2_) and remove photosynthetically generated O_2_, functions that are generally easier to manage at small scale because of shorter transport distances and greater mixing homogeneity. In *Chlorella vulgaris*, dissolved O_2_ concentrations of approximately 30–31 mg L^−1^ reduced biomass productivity by about 30%. This limitation was configuration-dependent: tubular PBRs and raceways were particularly susceptible to O_2_ accumulation under insufficient gas–liquid mass transfer, whereas airlift systems with kLa > 10 h^−1^ were predicted to avoid inhibitory accumulation. Correcting the limitation, however, created an opposing constraint. In a torus PBR, gas-flow rates of at least approximately 50 mL min^−1^ were required to control dissolved O_2_, whereas rates above approximately 100 mL min^−1^ promoted CO_2_ limitation through excessive degassing [48]. Increasing gas flow therefore did not eliminate the mass-transfer bottleneck; it narrowed operation to a configuration-specific window between O_2_ accumulation and carbon loss. This response was confirmed quantitatively in two strains selected for outdoor cultivation. In laboratory oxygen-response assays, increasing dissolved O_2_ from 0 to 30 mg L^−1^ reduced photosynthetic rates by 36.6% in *Monoraphidium minutum* 26B-AM and 26.1% in *Scenedesmus obliquus* UTEX393 [49]. When mitigation was subsequently tested in 100-L outdoor raceways, air sparging lowered dissolved O_2_ by 10–20 mg L^−1^ and increased *S. obliquus* areal biomass productivity by 36%, from 13.9 ± 0.4 to 18.9 ± 1.6 g m^−2^ d^−1^. No significant productivity improvement was observed for *M. minutum* during cold-season cultivation, demonstrating that removing O_2_ does not overcome other environmental limitations.

The interaction between gas balance and light further amplifies this limitation. Under a supersaturating irradiance of 900 µmol m^−2^ s^−1^, photosynthetic activity in *C. vulgaris* increased dissolved O_2_ to approximately 30 mg L^−1^ within 30 min. The resulting hyperoxic stress approximately doubled reactive oxygen species formation and reduced photosynthetic O_2_ evolution by about 70%, eventually causing bleaching and growth stagnation [50]. In contrast, removing O_2_ through N_2_ sparging prevented this response, confirming that the failure resulted from an imbalance between photosynthetic O_2_ generation and reactor stripping capacity. However, N_2_ sparging under controlled experimental conditions demonstrates the mechanism rather than providing a directly scalable remedy, because continuous gas replacement would introduce additional material and energy requirements. Thus, increasing irradiance can raise potential photosynthetic activity while simultaneously exceeding the reactor’s capacity to preserve an acceptable cellular environment.

CO_2_ supply creates the opposite but equally important constraint. Atmospheric CO_2_ (~0.04%, *v*/*v*) provides insufficient driving force for intensive biomass production, yet the efficiency with which enriched gas streams are retained remains low. Losses of supplied CO_2_ can reach 97% in open ponds, while minimum losses of approximately 25% in optimized closed PBRs and 50% in open ponds have been reported [12]. Consequently, increasing aeration or mixing to improve O_2_ stripping can simultaneously accelerate carbon loss and increase energy demand. In an approximately 2-L stirred-tank PBR, oxygen-balanced mixotrophic cultivation of *Chlorella sorokiniana* avoided external gas–liquid exchange by coupling respiratory O_2_ consumption with photosynthetic O_2_ production and using metabolically generated CO_2_ for carbon assimilation. Biomass concentration and reactor productivity approximately doubled relative to the photoautotrophic reference [12]. This result demonstrates the benefit of tightly balancing cellular production and consumption of O_2_ and CO_2_, but it also exposes the scale dependence of the solution: the metabolic and mixing control achieved in a 2-L reactor cannot be assumed to persist under outdoor conditions, spatial heterogeneity, and industrial transport distances.

Carbon availability must also be interpreted relative to nitrogen supply rather than as an isolated cultivation variable. In outdoor 1.0-m^3^ pilot-scale high-rate algal ponds treating blended wastewaters, increasing the influent C/N ratio from 9.65 to 25.31 and 52.71 reduced biomass productivity from 2.35 to 1.96 and 1.63 g VSS m^−2^ d^−1^, respectively, while carbohydrate content increased to 18.44% and 21.09% at the two higher ratios [51]. Thus, increasing carbon availability relative to nitrogen did not generate a universally superior outcome; it shifted the system from biomass formation toward carbohydrate accumulation and altered community composition.

An additional layer of complexity arises when the evaluation indicator shifts from biomass to the target product. The assumption that higher biomass productivity equates to higher product yield rarely holds under industrial conditions. Nitrogen deprivation redirects assimilated carbon from growth-associated processes toward storage compounds such as neutral lipids, but simultaneously constrains cell growth and division [52,53]. Consequently, increasing cellular lipid content does not necessarily increase lipid output per unit reactor volume or cultivation time. In outdoor flat panel PBR cultivation of *N. oculata*, moderate nitrogen limitation during spring increased lipid productivity from 0.11 to 0.21 g L^−1^ d^−1^, without an equivalent loss of biomass productivity [54]. However, the same advantage was not reproduced under the autumn cultivation conditions, demonstrating that the response depended on the interaction between nutrient supply, irradiance, season, and harvesting regime rather than on nitrogen limitation alone.

A complementary outdoor study demonstrates why this response cannot be generalized as “more nitrogen stress produces more lipid.” In 110-L Green Wall Panel PBRs, nitrogen deprivation increased the lipid content of *Nannochloropsis* sp. F&M-M24 from approximately 30% to more than 60% and initially increased lipid productivity from 117 ± 28 mg L^−1^ d^−1^ to 204 ± 47 mg L^−1^ d^−1^ [55]. Under progressively stronger limitation, however, biomass productivity declined and further increases in cellular lipid content did not consistently produce proportional gains in lipid productivity. The response therefore followed an intensity- and time-dependent optimum rather than a monotonic relationship. Optimizing cellular composition, biomass formation, and product output are distinct objectives, and a nutritional regime selected for one cannot be assumed to optimize the others.

From this point of view, the direct consequence is that optimizing a system for biomass and optimizing it for output are distinct objectives, and the choice between them is not merely biological. This choice determines which reactor geometry, which nutritional strategy, and which operational conditions are most appropriate. Thus, hydrodynamics completes the triangle of interdependencies. Insufficient mixing perpetuates light and gaseous gradients, while excessive mixing imposes mechanical shear that compromises more fragile cells and increases operational energy consumption [56,57]. In *Phaeodactylum tricornutum*, increased circulation power improved light exposure and biomass productivity up to a threshold; however, high shear rates induced cell stress, and values above approximately 30–80 s^−1^ caused severe culture damage [58]. In *Nannochloropsis oceanica*, increasing the raceway circulation velocity from 0.15 to 0.30 m s^−1^ increased areal biomass productivity by 1.7 g m^−2^ d^−1^ and resulted in an approximately 33% higher lipid content, but also doubled specific power consumption. Conversely, the lower velocity produced a slightly higher lipid-production-to-energy-consumption ratio, 17.1 versus 16.9 kg kW^−1^, and was therefore considered more suitable for energy-efficient lipid production under the evaluated conditions [59]. Thus, the hydrodynamic conditions that maximized biomass productivity or lipid content did not maximize energy-normalized lipid production. This dissociation illustrates why hydrodynamic optimization based on a single performance indicator can be misleading.

Large-scale hydrodynamic and environmental heterogeneity is compounded by the risk of microbial contamination, particularly in open cultivation systems. In commercial 130,000-L *Chlorella* ponds, infection by the predatory bacterium *Vampirovibrio chlorellavorus* was associated with the collapse of four cultivation units; bacterial attachment increased from approximately 20% to 100% within three days, followed by biomass decline and culture failure within 2–5 days. A targeted pH-shock treatment increased average culture longevity from 7.0 ± 2.7 to 12.7 ± 2.7 days [60]. Fungal parasites are similarly relevant at production scale: in outdoor *Graesiella* sp. raceways of up to 1000 m^2^ and 200,000 L, treatment with 10 mg L^−1^ SDBS prevented fungal infection without measurable penalties to biomass production or lipid content [61]. These examples illustrate how cell–contaminant interactions occurring at the microscale can cause the rapid loss of an entire production-scale culture. However, both interventions were validated for specific host–contaminant combinations and therefore represent targeted control strategies rather than universally transferable solutions to large-scale contamination.

In closed systems, this tension deepens for structural reasons. Tubular and flat-panel PBRs impose substantially more intense shear regimes on the cells than open systems, due to the forced pumping required to maintain adequate circulation and gas transfer. In *L. platensis*, the use of a centrifugal pump in a pilot system fragmented more than 90% of the filaments in less than 24 h, while volumetric productivity remained approximately three times higher than that observed in the raceway [62]. This result is informative not simply as an indication of productive performance, but as evidence of a dissociation between conventional productivity metrics and the physiological condition of the biomass. In other words, the system showed high volumetric productivity even under intense filament fragmentation, making it clear that conventional productivity metrics can mask physiological and morphological changes relevant to the long-term stability of the culture. In *Klebsormidium* cf. *nitens*, exceptional mechanical tolerance allowed stable growth even under high shear in tubular PBRs [63]. This reinforces the central argument that the viability of a closed PBR critically depends on the mechanical tolerance of the cultivated species, a variable rarely considered in strain-selection criteria for scale-up.

Ultimately, biological productivity must be distinguished from process-level energetic viability. In a pilot-scale continuous microalgal–bacterial PBR treating wastewater, the net energy ratio was 4.95–8.38 when the system boundary was limited to biomass production but decreased to only 0.14–0.23 when lipid extraction and biodiesel conversion were included; downstream operations accounted for approximately 88–90% of cumulative energy demand [64]. Therefore, improvements in reactor productivity or upstream energy efficiency do not necessarily produce a positive net energy balance when harvesting and conversion are considered. Net energy performance must consequently be evaluated across the integrated process using clearly defined system boundaries.

Anaerobic digestion must be distinguished according to its position within the production process. In integrated microalgal systems, controlled anaerobic digestion is generally applied downstream to harvested biomass or residual process streams rather than as part of the phototrophic cultivation stage. Pilot-scale HRAP studies have reported methane yields of 189–225 mL CH_4_ g^−1^ VS from mono-digestion of microalgal biomass and 238–258 mL CH_4_ g^−1^ VS when the biomass was co-digested with primary sludge [65]. However, these methane yields do not by themselves demonstrate a positive process-level energy balance, because biomass harvesting, concentration, pretreatment, digestion, and biogas utilization must also be included within the system boundary [64,65]. Taking together, these cultivation- and process-level constraints become particularly evident when their quantitative effects are compared across different systems and validation scales. Table 1 summarizes representative evidence obtained from controlled laboratory experiments, modeling analyses, pilot, outdoor and industrial-scale systems, selected according to its relevance to production-scale cultivation.

Taken together, these results highlight a recurring paradox in microalgal scaling, where interventions available to correct one limitation tend to intensify another, and the system responds not to the isolated variable that was manipulated, but to the new state of disequilibrium that this manipulation created. Increasing cell density increases volumetric productivity but intensifies self-shading and deepens CO_2_ and O_2_ gradients; increasing irradiance raises photosynthetic potential but favors photoinhibition and accelerates the accumulation of dissolved oxygen to inhibitory levels; intensifying mixing reduces light and gaseous gradients but increases mechanical shear and operational energy consumption; and inducing nutritional stress increases the content of the target product but compresses the productivity of the biomass that carries it.

The thermal gradient permeates all these relationships; systems that control temperature to sustain cellular physiology incur costs that compromise the economic viability of the biological gains obtained. Scaling up, therefore, does not consist of maximizing isolated variables, but in managing the trade-offs generated by the interaction between them, and the central difficulty is not technical, but epistemic: models built from laboratory cultures, where each variable can be controlled individually, do not capture the interdependence that defines the behavior of the system when all operate simultaneously, at scale, under varying environmental conditions. This scheduling paradox is conceptually summarized in Box 1, which illustrates how optimizing a single operational variable frequently generates unintended constraints in other dimensions of the system.

Box 1 **The scheduling paradox: when optimizing one system function destabilizes another.**

**Figure 2:**
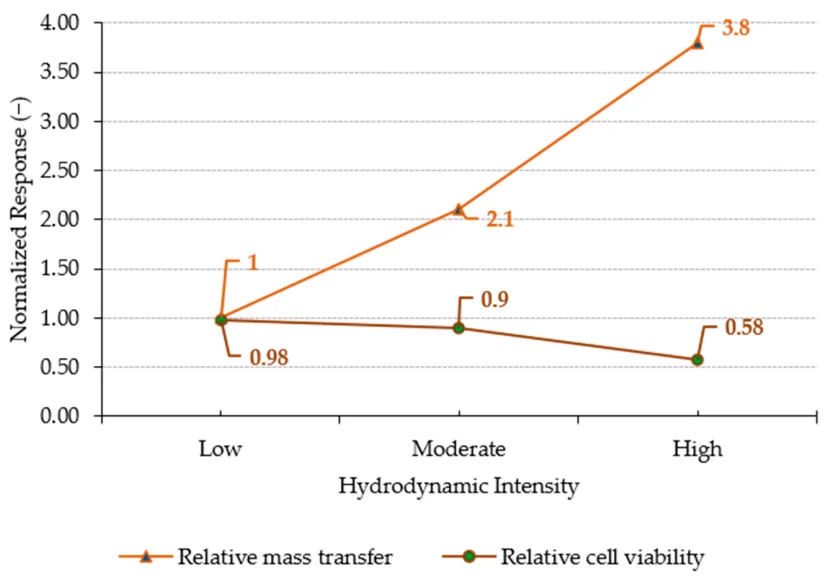
Conceptual illustration of the trade-off between increasing hydrodynamic intensity, mass transfer, and cell viability in microalgae cultivation systems.

**Figure 3:**
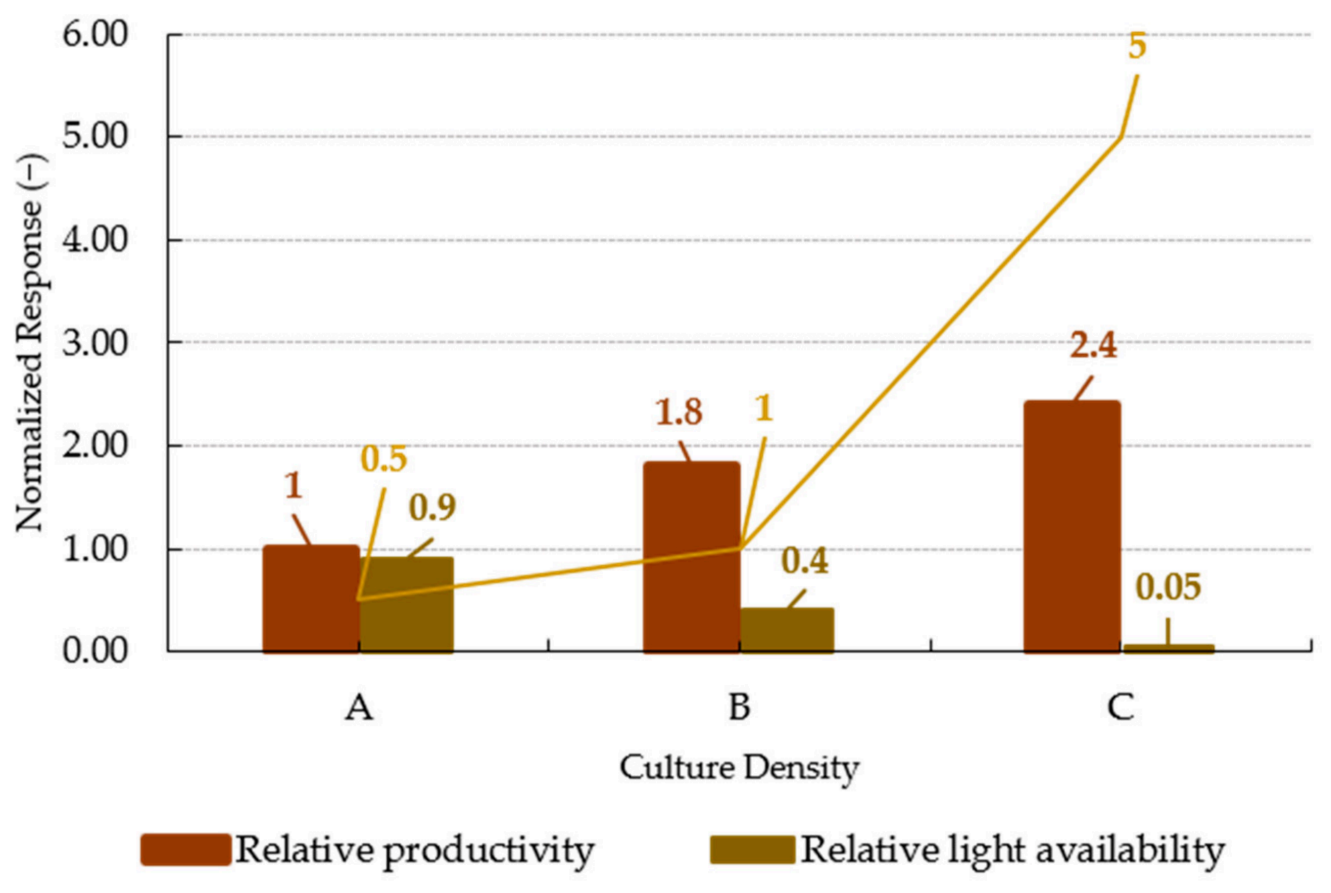
Conceptual illustration of the trade-off between increasing culture density, volumetric productivity, and light availability in microalgae cultivation systems.

Operational intensification in microalgal cultivation systems rarely produces one-dimensional gains. The interactions between mixing, cell density, light availability, and gas transfer create a field of trade-offs in which each optimized variable exerts pressure on the others. Figure 2 and Figure 3 conceptually illustrate two of these non-linear relationships, between hydrodynamic intensity and cell viability, and between culture density and photosynthetic efficiency, constructed based on trends consistently reported in the literature [13,32,62,67]. The values are relative and schematic; their function is not to reproduce a specific experimental system, but to make the structural pattern visible: on an industrial scale, optimizing one dimension of the system almost invariably means degrading another.

Increased mixing and aeration generally promote CO_2_ transfer, nutrient distribution, and crop homogenization. However, increased turbulence also raises hydrodynamic shear stress, which can compromise membrane integrity and reduce cell viability in shear-sensitive microalgae species. Thus, the same mechanism that promotes mass transfer and reduces environmental gradients can compromise cellular integrity when it exceeds a physiological tolerance threshold. Similarly, increasing biomass concentration generally raises volumetric productivity due to the greater number of cells present in the culture. However, higher cell densities intensify self-shading and internal light gradients, reducing light penetration into the deeper regions of the photobioreactor. Consequently, although denser crops contain more biomass, productivity gains become progressively non-linear as photosynthetic efficiency decreases under light limitation. Conversely, the same mechanism that increases volumetric productivity also reduces the availability of light per cell, thereby establishing a physiological limit to the benefits of increased cell density. Taken together, these examples demonstrate that improving microalgal cultivation performance cannot rely on the isolated optimization of individual variables. Instead, effective scale-up requires balancing competing physiological and engineering constraints that emerge simultaneously within the production system.

## 4. Microspace Bottlenecks: The Cell as a Target for Engineering

To shift our perspective from macro to micro scale, we need to understand that the performance of large-scale microalgae cultivation systems depends not only on the physical efficiency of the reactor, but also on the cells’ ability to respond physiologically to the environmental fluctuations imposed by that reactor. On an industrial scale, each cell follows distinct environmental paths, being subjected to intermittent light oscillations, CO_2_ and O_2_ gradients, pH fluctuations, nutritional restrictions, and hydrodynamic shear. At the microscopic scale, these environmental fluctuations affect not only the instantaneous photosynthetic rate but also how the cell distributes its energy resources.

Under conditions of stress or limited resources, investment in growth and biomass accumulation may be reduced in favor of activating defense, repair, and adaptation mechanisms. As a result, cell-cycle regulation emerges as one of the most critical microspatial bottlenecks linking environmental fluctuations to culture productivity. In microalgae, growth and cell division are strongly influenced by environmental conditions and are often synchronized with light/dark cycles, rather than occurring continuously or homogeneously [68,69]. Factors such as light availability, carbon assimilation, intracellular energy status, and nutrient availability determine not only the instantaneous photosynthetic rate but also the duration of the G1 phase, the principal growth phase in which cells accumulate carbon, biomass, and energy reserves before committing to division; the critical size required for division; and the proliferation frequency.

**Figure 4:**
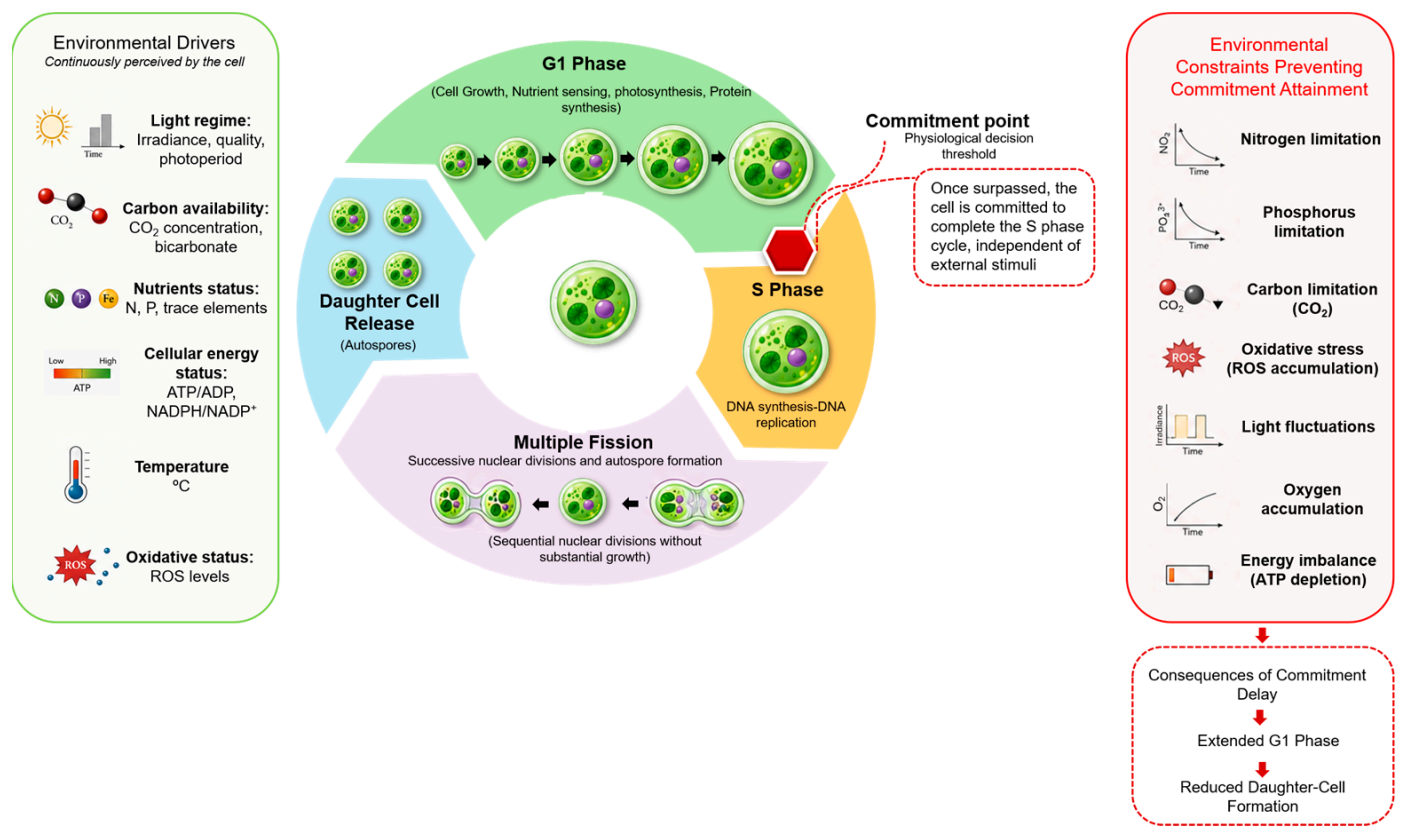
Conceptual representation of the environmental and metabolic regulation of the micro-algal cell cycle. The cycle proceeds clockwise from the G1 growth phase, during which cells increase in size and integrate environmental, nutritional, energetic, and redox signals, through attainment of the commitment point, DNA replication during S phase, successive nuclear divisions and autospore formation during multiple fission, and daughter-cell release; the released daughter cells subsequently re-enter G1. The red hexagon indicates the commitment point, a physiological threshold after which the cell is committed to completing the division program. The left panel summarizes environmental and intracellular drivers continuously perceived by the cell, whereas the right panel presents constraints that can delay or prevent commitment-point attainment. The downward red arrows indicate the consequences of delayed commitment, including extension of G1 and reduced daughter-cell formation. Abbreviations: G1, first gap or growth phase; S, DNA-synthesis phase; DNA, deoxyribonucleic acid; N, nitrogen; P, phosphorus; CO_2_, carbon dioxide; O_2_, oxygen; ROS, reactive oxygen species; ATP, adenosine triphosphate; ADP, adenosine diphosphate; NADPH, reduced nicotinamide adenine dinucleotide phosphate; NADP^+^, oxidized nicotinamide adenine dinucleotide phosphate; °C, degrees Celsius. Original conceptual illustration created by the authors based on the cell-cycle and environmental-regulation mechanisms described in [68,72,74,75,76].

In *Chlamydomonas reinhardtii*, the G1 phase can last approximately 10 to 14 h, while subsequent S/M subcycles occur in just 30–40 min [70]. The commitment point in the cell cycle is the critical “point of no return” during the G1 phase. Once the cell surpasses this point, it is obliged to complete the entire cell division cycle, regardless of external stimuli; it is not a fixed event, but a threshold dynamically modulated by energy state, carbon availability, and light regime [71,72,73]. Consequently, small changes in environmental conditions throughout the G1 phase can prevent the product from reaching its target and directly reduce the final number of daughter cells generated. Checkpoints reinforce this sensitivity, restricting subsequent splits in situations of energy restriction, carbon limitation, or excessive oxidative stress. As illustrated in Figure 4, progression through the microalgal cell cycle depends on the continuous integration of environmental and metabolic signals, which modulate the duration of the G1 phase and the probability of reaching the commitment point.

This dynamic becomes even more relevant given the diversity of proliferative strategies observed among microalgae. While some species perform only one binary division, generating two daughter cells per cycle, others are capable of performing multiple divisions and simultaneously forming several autospores within a single mother cell [74,75]. In species of the genera *Chlorella* and *Scenedesmus*, for example, a single cell can give rise to 2, 4, 8, or even 16 autospores in the same cell cycle. This pattern results from a prolonged period of growth in G1, during which the cell accumulates biomass, carbon, and energy reserves until it reaches the critical size necessary to trigger successive rounds of nuclear replication and cytokinesis [68,77].

Consequently, any environmental limitations experienced during this phase can compromise the achievement of this physiological threshold, reducing the number of subsequent divisions and, ultimately, the productivity of the crop. Thus, even cells belonging to the same population and cultivated under the same average operating conditions may exhibit distinct physiological trajectories due to microenvironmental differences experienced over time. The result is a physiologically heterogeneous population, in which growth, cell division, and productivity cease to be uniform characteristics and begin to reflect the individual environmental history of each cell.

Nutritional status also directly interferes with this proliferative dynamic. In simplified binary fission models, nutrients such as nitrogen and phosphorus are usually treated only as general metabolic substrates [77]. In multi-division microalgae, however, these elements act as indirect regulators of the cell cycle itself [68,70]. Nitrogen deficiency often restricts growth and protein synthesis while redirecting metabolism toward storage compounds such as lipids and carbohydrates. Phosphorus limitation directly interferes with processes associated with ATP and phospholipid synthesis and DNA replication, affecting cell progression and proliferative capacity [31,78,79]. In *Chlorella sorokiniana*, Ca^2+^ depletion was associated with growth stagnation and blockage of normal multiple fission progression, promoting the accumulation of cells in intermediate autosporic stages, demonstrating that cell division does not depend solely on a fixed internal program or light availability, but on the continuous interpretation of the local chemical environment perceived by the cell [78]. In industrial systems, where microgradients of nutrients, ions, and pH are unavoidable, this sensitivity directly contributes to the formation of subpopulations in different physiological states.

Light heterogeneity represents one of the clearest examples of this transfer from the macro to the microscale. In dense cultivation, self-shading reduced light penetration while the mixture continuously shifts cells between light and dark regions. In thin-layer cascade reactors, individual cells can experience light/dark cycles between 0.1 and 2 s, while phototactic responses and acclimation mechanisms operate on scales of tens of seconds, resulting in a decoupling between the physical time of the reactor and the biological time of the cell. As a consequence, a significant portion of the absorbed energy is no longer directed towards carbon assimilation and is instead dissipated in photoprotective and oxidative repair mechanisms, reducing the availability of ATP and NADPH for biomass synthesis, prolonging the G1 phase, and delaying the start of cell division [80]. The ultimate impact is not just lower instantaneous photosynthetic efficiency but a cumulative reduction in population productivity.

Gas transfer bottlenecks follow a similar logic. In dense systems, local CO_2_ gradients coexist with the accumulation of dissolved O_2_, favoring photorespiration and the formation of reactive oxygen species (ROS) [52,53]. Under these conditions, a significant portion of photosynthetic energy is no longer directed towards growth and instead supports antioxidant mechanisms and cellular repair [76,81].

The problem, therefore, lies not only in the average availability of inorganic carbon but in the microenvironmental instability with which this carbon reaches the cells. Hydrodynamic stress represents the final link in this chain: strategies used to intensify mixing and reduce gradients simultaneously increase the mechanical shear imposed on cells, raising structural damage, wear, and energy costs associated with cell repair [11,71,81]. Under stress, many microalgae increase the secretion of extracellular polymeric substances (EPS), altering the viscosity and mass transfer of the medium, feeding back into the hydrodynamic stress and making the bottleneck simultaneously physical and biological [82,83].

Classical growth models generally assume homogeneous populations and continuous growth, neglecting the fact that each cell experiences distinct trajectories of light, nutrients, and mechanical stress [7,8]. As a result, actual productivity often diverges from that predicted by average operating parameters. The real bottleneck emerges when the physical dynamics of the system become incompatible with the physiological timelines of the cell, and productivity begins to depend not only on average cultivation conditions but also on the system’s ability to maintain metabolically synchronized and physiologically stable populations in the face of the inevitable heterogeneity of industrial scale.

Taken together, this evidence demonstrates that microspatial bottlenecks do not emerge from an isolated physiological limitation but from the continuous interaction between the physical dynamics of the system and the cellular capacity to respond to it. Changes in the availability of light, nutrients, inorganic carbon, dissolved oxygen, and mixing intensity directly affect cell cycle regulation, energy allocation, and metabolic stability of populations. The central challenge, therefore, lies not only in the biological potential of the cell lines but in the incompatibility between the speed of fluctuations imposed by industrial cultivation and the cellular capacity to respond to them in a coordinated and productive manner. In other words, the problem lies not exclusively in the cell or the reactor, but in the lack of synchronization between the two.

Given this diagnosis, a fundamental question arises: would it be possible to engineer the cell itself to respond to this environment in a more robust, resilient, and productive way?

### Can Bioengineering Reduce Cellular Fragility?

The microspatial bottlenecks discussed earlier demonstrate that many of the limitations observed in industrial culture systems arise from the continuous interaction between the cell and a highly heterogeneous environment. Fluctuations in light, nutrients, dissolved gases, and shear directly affect fundamental physiological processes, including growth, photosynthesis, cell cycle regulation, and allocation of metabolic resources.

Given this scenario, a central question arises: to what extent is cellular bioengineering capable of reducing this physiological vulnerability? While the spatiotemporal heterogeneity inherent in production-scale cultivation can hardly be completely eliminated, a frequently proposed alternative is to develop strains capable of better tolerating these fluctuations and maintaining productive performance under variable environmental conditions. The question that organizes this section is therefore twofold: what can cellular bioengineering already do about these bottlenecks, and where are the limits of these interventions when applied outside the laboratory conditions that generated them?

Cellular bioengineering and strain-improvement strategies in microalgae have advanced along at least five fronts directly relevant to the cellular bottlenecks identified above. The first involves improving growth, carbon assimilation, and inorganic-carbon acquisition. Studies targeting RuBisCO activation and bicarbonate transport demonstrate that engineered gains can depend strongly on CO_2_ availability, indicating that the benefit of a modified phenotype cannot be evaluated independently of its cultivation environment [83,84].

The second front involves photosynthetic optimization through modifications in light-harvesting antenna size and non-photochemical energy dissipation. Although these strategies can improve biomass formation under high irradiance or in dense cultures, their benefits depend on the optical environment, including irradiance, culture depth, and cell density. The maintenance of a strain-improvement advantage under outdoor cultivation, as demonstrated for the TAM-2 mutant of *Chlorella sorokiniana*, remains uncommon and is therefore particularly relevant to the translation of photosynthetic optimization beyond controlled laboratory conditions [85].

The third front involves redirecting carbon toward target products through the modification of transcriptional regulators and lipid-biosynthesis pathways. These interventions demonstrate the technical capacity to increase lipid content and productivity and, in some cases, to redirect carbon from carbohydrates toward lipids [86,87,88,89]. However, most evidence remains confined to controlled cultivation conditions, and the translational significance of these phenotypes depends on whether their metabolic advantages remain stable under the environmental fluctuations and operational stresses characteristic of production-scale cultivation.

A fourth front involves increasing tolerance to oxidative stress. In *Nannochloropsis salina*, heterologous expression of the cyanobacterial alcohol-dehydrogenase gene *sysr1* reduced intracellular ROS accumulation and increased the activities of superoxide dismutase, ascorbate peroxidase, and catalase under cold stress, improving physiological tolerance relative to the wild type [90]. A fifth front concerns the mechanical robustness of cells exposed to the physical conditions imposed by large-scale cultivation. Although frequently overlooked, this characteristic can determine whether gains achieved through cellular engineering are maintained during scale-up and whether the resulting biomass remains compatible with downstream processing [63,81,91]. Representative quantitative outcomes for the first three engineering fronts are compared in Table 2 together with their experimental context and translational relevance.

Taken together, the quantitative evidence summarized in Table 2 indicates that cellular bioengineering and strain-improvement strategies have the technical capacity to mitigate several of the microspatial bottlenecks identified above but also reveals a pronounced asymmetry between the magnitude of the reported gains and their level of translational validation. Improvements approaching or exceeding 30–100% are achievable for biomass- or product-related traits, yet most remain demonstrated predominantly under controlled laboratory conditions. Even when a phenotype is maintained outdoors, as observed for antenna reduction in *C. sorokiniana*, its advantage remains dependent on the environmental context in which it is expressed. The problem therefore arises when growth, physiological stability, and operational robustness enter the equation simultaneously. This reveals one of the central paradoxes of bioengineering in microalgae: increasing product accumulation does not necessarily mean increasing the actual productivity of the process. In some cases, metabolic redirection toward storage compounds may alter cellular energy allocation, oxidative balance, or physiological flexibility under environmental fluctuations. Metabolic gain, therefore, does not eliminate the microspatial bottleneck; it merely shifts physiological priorities within the cell. It is at this point that mechanical robustness engineering emerges not as an alternative to other strategies, but as a complementary requirement for maintaining their benefits under production-relevant hydrodynamic conditions.

It is worth noting that the mechanical fragility of microalgae in industrial systems is not a uniform phenomenon. Tolerance to hydrodynamic forces is highly species-specific and is largely determined by cell wall composition and architecture. In *Nannochloropsis* spp., a thick cell wall composed of algaenan, cellulose, and hemicellulose, with a thickness ranging from 63 to 119 nm depending on the strain [101], provides remarkable mechanical resistance. This robustness is primarily attributed to the outer algaenan layer, an insoluble aliphatic biopolymer that is highly resistant to physical disruption [91], enabling these species to withstand the operational conditions commonly encountered in industrial photobioreactors. In contrast, strains with reduced or absent cell walls, such as cw-type *C. reinhardtii* mutants, exhibit pronounced sensitivity to hydrodynamic forces, displaying measurable cell mortality at superficial gas velocities that do not affect wild-type strains [63]. As a result, these mutants are poorly suited to cultivation systems requiring intense mixing and aeration.

In this context, this structural difference reveals something more interesting than a taxonomic distinction: the selection of strains for industrial cultivation has predominantly relied on metabolic criteria, while mechanical robustness has often been treated as a secondary attribute and evaluated only during scale-up.

From this, cell wall engineering emerges as an approach with dual and often opposing implications. On the one hand, the intentional weakening of the cell wall, as demonstrated by the CRISPR/Cas9 deletion of the CesA1 cellulose synthase gene in *Nannochloropsis salina*, increased lipid extractability by 1.7-fold [102]. However, weakening the cell wall may increase susceptibility to mechanical and oxidative stresses, creating a potential trade-off between cultivation robustness and downstream extractability. Conversely, strategies aimed at strengthening the cell wall or modulating stress-responsive structural proteins can increase cell viability under shear stress without necessarily compromising product accumulation.

The engineering of cell wall-anchored proteins, as demonstrated in glycomer- and intein-pH-based systems in *C. reinhardtii* [103], highlights how targeted structural modifications can improve cellular resilience to mechanical stress, expanding physiological tolerance while preserving the strain’s metabolic performance.

This duality exposes a fundamental limitation of cell wall engineering as a standalone strategy: the same structural properties that provide mechanical protection in bioreactors often hinder downstream processing, and vice versa. No single modification of the cell wall is capable of simultaneously addressing the challenges associated with both cultivation and industrial processing. Similarly to the metabolic engineering strategies discussed previously, structural interventions tend to solve one bottleneck at the cost of intensifying another. Cells with more robust cell walls generally require greater energy input for cell disruption, whereas cells with weakened cell walls become more susceptible to the physical stresses imposed by large-scale cultivation.

This limitation does not invalidate cellular bioengineering. On the contrary, it demonstrates its relevance as a tool for enhancing the physiological robustness of microalgae and mitigating some of the microspatial bottlenecks discussed previously. However, it also highlights that industrial performance does not depend exclusively on the genetic characteristics of the strain, but rather on the continuous interaction between these characteristics and the dynamic conditions of the cultivation environment. Thus, although cellular engineering can improve growth, stress tolerance, photosynthetic efficiency, and the accumulation of target products, the translation of these gains into industrial systems remains dependent on the ability of the cultivation system to sustain these advantages over time and under variable operating conditions.

Taken together, these findings point to a fundamental limitation of cellular bioengineering as a standalone strategy: genetic interventions that are successful under laboratory conditions do not necessarily guarantee equivalent performance at the industrial scale, because the bottleneck lies not only within the cell itself, but also in the relationship between the cell and the dynamic environment in which it operates. This observation does not invalidate the approaches discussed above; rather, it reframes the problem. The relevant question is no longer exclusively how to make the cell more efficient, but under which conditions this efficiency can be sustained as cultivation systems scale up. This question, which cellular bioengineering alone cannot fully answer, provides the starting point for the next section.

## 5. Precision Microalgae: When the System Learns from the Cell

The conventional paradigm of microalgal biotechnology seeks to reduce system variability through the control of cultivation conditions. The *precision microalgae* paradigm is based on a different premise: heterogeneity is an inherent feature of complex biological systems and, therefore, must be monitored, interpreted, and incorporated into the decision-making process. Rather than optimizing reactors, strains, or operational strategies in isolation, this approach aims to continuously integrate information from the cell, the population, and the cultivation system into a cycle of observation, interpretation, prediction, and response.

This shift in perspective emerges directly from the bottlenecks discussed in the previous sections. Although significant advances have been achieved in photobioreactor engineering, cellular bioengineering, and process monitoring, most production systems still operate based on average values, assuming that the culture responds homogeneously to the conditions imposed by the reactor. Individual cells, however, do not respond to operational averages; they respond to the local conditions they experience over time. Consequently, the challenge is no longer limited to controlling process variables but extends to understanding how these variables are perceived by the cell and how they influence its physiological and metabolic behavior.

The body of evidence presented in the previous sections converges on a common diagnosis: the primary limitation of industrial microalgal biotechnology is not the lack of metabolically capable cells, but the inability of current systems to monitor and respond, in real time, to the environmental fluctuations that occur during cultivation. Photobioreactor engineering has reduced physical gradients, cellular bioengineering has expanded the metabolic capabilities of microalgae, and strategies aimed at improving physiological robustness have enhanced tolerance to a range of environmental stresses. Nevertheless, most production systems still operate based on average process values, assuming that cultures respond homogeneously to the conditions imposed by the reactor, a premise that the preceding sections have shown to be systematically flawed.

The analogy with precision agriculture is not merely rhetorical, but structural. Just as precision agriculture replaced uniform prescriptions with strategies based on the continuous observation of spatial variability across the field, *precision microalgae* propose replacing static operational strategies with adaptive decisions informed by the real-time state of the culture. In agricultural systems, this approach has enabled adjustments in seeding density, irrigation, and fertilization according to local productivity, transforming heterogeneity into actionable information for decision-making. In microalgal cultivation, the principle is the same: the objective is not to create a uniformly optimal environment for all cells, but rather to characterize heterogeneity with sufficient resolution so that interventions related to light, nutrients, carbon supply, pH, or harvesting can be continuously adjusted according to the observed biological response. The unifying principle is not the cultivation system itself, but the decision-making logic, namely, replacing fixed recipes with strategies capable of continuously learning from the biological system.

Operationally, *precision microalgae* can be understood through three complementary pillars: (i) monitoring, responsible for capturing the physiological state of the culture through sensors and continuous data acquisition; (ii) prediction, based on computational models, digital twins, and artificial intelligence to interpret and anticipate system behavior; and (iii) adaptation, in which control strategies and bioengineering approaches translate this information into interventions capable of adjusting cultivation conditions in response to the state of the culture. The examples discussed below illustrate how these pillars are beginning to emerge in the current literature and why their integration represents a conceptual shift from conventional approaches.

**Figure 5:**
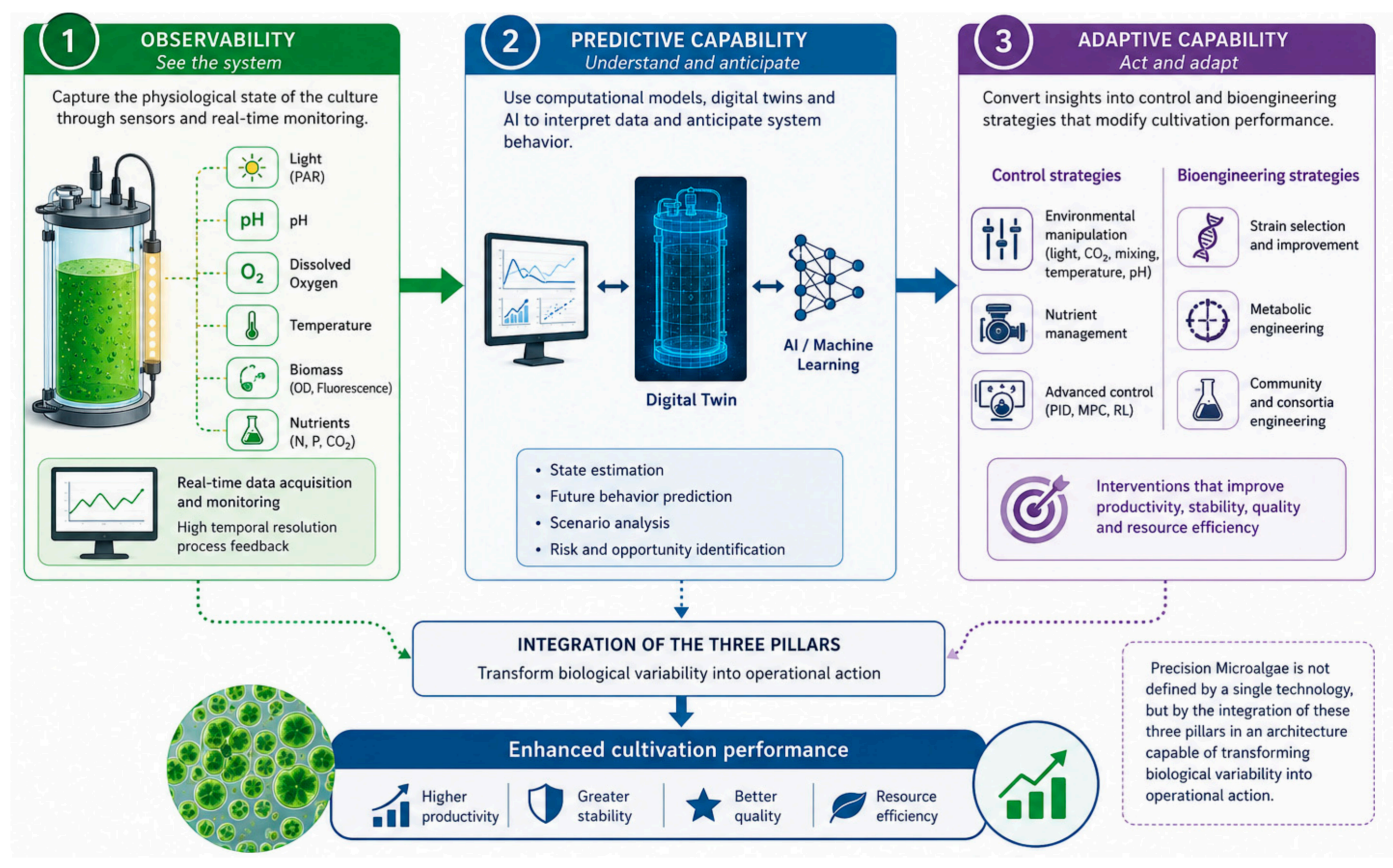
*Precision microalgae* as an integrated framework for biologically informed cultivation management.

The conceptual framework proposed for *precision microalgae* is summarized in Figure 5, where real-time monitoring, predictive capabilities, and adaptive control are integrated into a continuous decision-making cycle that transforms biological variability into operational actions.

Among the variables most frequently monitored are light intensity, biomass concentration, optical density, pH, temperature, dissolved oxygen, CO_2_, and nutrients, particularly nitrogen and phosphorus. The importance of such monitoring stems from the fact that microalgal physiology depends not only on the average values of these variables, but also on the dynamic exposure history experienced by cells. In other words, two cultures may exhibit the same average irradiance or nutrient concentration yet respond in entirely different ways if they have been exposed to distinct environmental trajectories throughout cultivation. Consequently, systems capable of continuously tracking these changes become strategic tools for reducing physiological fluctuations and increasing process predictability.

The most significant advances have been observed in intelligent light-control strategies. In conventional systems, irradiance remains fixed throughout cultivation, regardless of cell density or the physiological state of the culture. As biomass accumulates, self-shading alters the light availability perceived by individual cells, significantly affecting photosynthetic efficiency. Reference [104] implemented a lumostatic strategy based on image analysis and chlorophyll *a* monitoring in *Chlorella* sp., continuously adjusting light intensity between 82 and 590 μmol m^−2^ s^−1^ according to culture development. Maximum biomass reached 5.78 g L^−1^, and productivity achieved 1.29 g L^−1^ d^−1^, representing increases of 25.7% and 74.3%, respectively, compared with the best constant-light condition. These results are consistent with the hypothesis that a substantial portion of photosynthetic efficiency losses does not arise from intrinsic metabolic limitations, but rather from a mismatch between light supply and the physiological requirements of the culture over time. It is important to note, however, that this experiment was conducted in a laboratory-scale draft-tube photobioreactor under optical and hydrodynamic conditions that were substantially more controlled than those encountered in large-scale outdoor systems. Whether these gains can be reproduced under industrial cultivation conditions, characterized by variable solar irradiance and more pronounced optical gradients, remains an open question.

The same principle has been applied to nutrient control. In *Chlorella vulgaris*, a closed-loop Genome-Scale Model Process Control (GMPC) system was used to predict glucose and nitrate consumption and automatically adjust reactor feeding. Compared with conventional fed-batch operation, the system reduced glucose consumption by 75% and nitrate consumption by 23%, increased biomass yield per unit of glucose consumed by 122% and reduced total control error by approximately 80% relative to traditional PID controllers [105]. Similar advances have been reported in pH and nitrogen control. In ammonium-rich systems, appropriate pH regulation reduced volatilization losses from 29.6–71.0% to less than 5%, while simultaneously increasing biological nutrient assimilation [106]. Beyond reducing input consumption, these results demonstrate that the integration of monitoring and predictive modeling can better align resource supply with the actual physiological demands of the cells. It should be emphasized, however, that both the GMPC system and the pH-control experiments were validated in 2-L bioreactors and pilot-scale cultivations, respectively. The transfer of these strategies to industrial-scale systems, where concentration gradients and operational variability are substantially greater, has yet to be systematically demonstrated.

In parallel, the integration of sensors and artificial intelligence has expanded the observational capabilities of cultivation systems. In instrumented closed tubular photobioreactors equipped with multiple sensors and cameras, XGBoost models achieved an R^2^ = 0.9997 for real-time growth prediction [24]. In low-cost systems based on turbidity and temperature sensors, correlations with dry weight reached R^2^ = values of 0.943–0.986, enabling automated biomass control for less than USD 330 [14]. Optical fiber sensors for pH and dissolved oxygen offer high sensitivity and the possibility of multiplexing across multiple locations within the reactor, making them promising tools for capturing intrareactor heterogeneity in high-density cultures [107].

These advances represent a significant achievement in observability, and this distinction matters. Most of these studies demonstrate the ability to accurately estimate what is occurring within the reactor; they do not yet demonstrate that this information has been translated into sustained productivity gains at the industrial scale. In precision agriculture, the historical sequence followed precisely this trajectory: first, it became possible to observe field variability with sufficient resolution; only then did it become possible to prescribe interventions based on that variability. In microalgal biotechnology, many systems remain at this first stage. Recognizing this reality does not diminish the progress achieved but rather places it honestly within the technological maturity trajectory of the field.

The second pillar of *precision microalgae* operates at the interface between reactor modeling and cellular modeling. Digital twins, dynamic computational representations of a physical system continuously updated with real-time data, have been proposed as tools for integrating photobioreactor hydrodynamics with microalgal growth and metabolic models [25,26]. Recent advances in genome-scale metabolic models (GEMs) for *C. vulgaris* and *Nannochloropsis* have brought this multiscale integration closer to reality by linking reactor-level parameters to intracellular metabolic fluxes [107]. In addition, the application of transfer learning to adapt hybrid mechanistic–predictive models to new strains using minimal datasets suggests that the cost of developing strain-specific digital twins can be substantially reduced [108].

A limitation that is rarely acknowledged explicitly in this literature, however, is that the vast majority of these models have been trained and validated under laboratory conditions. Their ability to capture the stochastic variability of industrial-scale outdoor systems, where solar irradiance, temperature, and medium composition fluctuate in an uncontrolled manner, remains one of the major unresolved challenges in the field. A model that is highly accurate at the bench scale may prove fragile under real-world operating conditions: the heterogeneity that a digital twin must ultimately manage at scale is precisely the heterogeneity that was absent from its training data.

The third pillar represents the convergence of genetic engineering and computational intelligence. The integration of machine learning with genome-editing technologies, including second- and third-generation CRISPR systems, such as CRISPRa/i for reversible transcriptional modulation and base editors for targeted modifications without double-strand DNA breaks, opens the possibility of data-driven strain design, in which predictive models identify intervention targets and anticipate system-wide consequences before editing is performed [107,108]. Machine-learning models have already demonstrated accuracy exceeding 90% in predicting lipid productivity from integrated cultivation parameters [90]. Looking further ahead, within the context of generative synthetic biology, synthetic regulatory circuits capable of modulating gene expression in response to environmental signals, such as ROS accumulation, CO_2_ limitation, or shear stress, represent a logical extension of this paradigm [109].

This area, however, exhibits the greatest gap between promise and demonstration. Predicting productivity under standardized laboratory conditions is not equivalent to controlling gene expression in variable industrial environments, and the long-term stability of synthetic circuits during extended cultivation remains an unresolved challenge, particularly in systems subject to selective pressures acting against the transgenes themselves.

The quantitative evidence supporting these three pillars is summarized in Table 3. Rather than presenting the reported improvements independently of their experimental context, the table compares the effect of each strategy with the scale at which it was validated. This distinction is essential because high monitoring accuracy, improved resource-use efficiency, or increased productivity under control conditions does not necessarily demonstrate that the same performance can be sustained under production-scale heterogeneity.

Taken together, these three pillars define what distinguishes *precision microalgae* from conventional approaches. The focus shifts away from the search for fixed optimal conditions and toward the ability of the system to continuously perceive, interpret, and respond to biological variability. Heterogeneity is no longer treated solely as a source of operational noise, but rather as a source of information capable of guiding cultivation decisions, control strategies, and strain-development efforts. In other words, *precision microalgae* do not represent a specific technology, but a paradigm shift: from systems designed to control the culture to systems designed to learn from it.

If precision agriculture transformed spatial heterogeneity into site-specific management, *precision microalgae* propose to transform physiological and operational heterogeneity into dynamic decision-making within the photobioreactor. As summarized in Table 3, this proposal is already supported by partial quantitative evidence demonstrating improvements in monitoring accuracy, resource-use efficiency, process control, and productivity. However, the validation scales reported in the table also show why *precision microalgae* cannot yet be considered a consolidated technological solution: most strategies remain confined to laboratory- or pilot-scale systems, with only isolated demonstrations under production-relevant conditions. The available evidence therefore supports the operational logic of the paradigm, but not yet its generalized industrial effectiveness. This distinction provides the critical context for the conceptual comparison presented in Box 2.

Box 2 **From Precision Agriculture to Precision Microalgae: A Shared Framework for Managing Biological Variability.**

The comparison between precision agriculture and precision microalgae should not be interpreted as a simple transfer of technologies between distinct sectors, but rather as the application of a common paradigm for managing biological variability. Precision agriculture became established through the recognition that spatial heterogeneity within agricultural fields contains agronomically relevant information and, therefore, should be monitored, quantified, and incorporated into decision-making processes [15,16]. Similarly, microalgal cultivation systems operate under intense temporal, spatial, and physiological heterogeneity arising from dynamic gradients of light, CO_2_, dissolved oxygen, pH, nutrients, and cellular metabolic state [4,6,7,8,10]. Just as sensors, yield maps, and predictive models transformed spatial variability into site-specific agronomic decisions, in-line sensors, artificial intelligence, mechanistic models, and digital twins are beginning to transform the physiological variability of microalgal cultures into actionable operational information. In this context, *precision microalgae* can be understood as the convergence of real-time monitoring, predictive modeling, adaptive process control, and targeted bioengineering, with the goal of improving productivity, robustness, and resource-use efficiency throughout the production process. Table 4 summarizes the conceptual correspondence between precision agriculture and precision microalgae.

Although these technologies remain at different stages of development, the comparison presented in Table 4 indicates that the principal structural elements of precision agriculture already have functional counterparts in microalgal cultivation. Whereas precision agriculture seeks to manage spatial variability across cultivated fields, precision microalgae aim to interpret and manage the physiological and environmental variability that continuously emerges within cultivation systems. This correspondence does not imply that the two fields have reached equivalent levels of technological maturity or production-scale validation. Instead, it provides a conceptual structure for identifying how monitoring, prediction, and adaptive intervention could convert biological heterogeneity into operational decisions in microalgal cultivation.

**Table 4:** From spatial variability to physiological variability: conceptual correspondence between precision agriculture and precision microalgae.

Precision Agriculture	Precision Microalgae
Yield maps and field history	Online estimation of biomass and culture state using turbidity, optical sensing, computer vision, and predictive models
Variable-rate application	Adaptive nutrient feeding, turbidostat operation, and dynamic cultivation setpoints
Field sensors and diagnostics	In-line monitoring of pH, dissolved oxygen, temperature, biomass, and culture state
Management-zone prescriptions	Adaptive light management, biomass control, and closed-loop cultivation strategies
Predictive models and yield forecasting	Mechanistic and data-driven models, hybrid modeling, and digital twins for predictive cultivation management
Cultivar selection according to local conditions	Selection and bioengineering of strains for productivity, robustness, and environment-specific performance
Phenological-stage management	Monitoring and management of cell-cycle progression, physiological state, stress responses, and harvest timing

Nevertheless, important limitations remain. Most of the quantitative evidence available to date still originates from laboratory- or pilot-scale studies conducted under optical, hydrodynamic, and nutritional conditions that are substantially more stable than those encountered in industrial outdoor systems. More importantly, demonstrating the ability to monitor, predict, or manipulate the state of a culture does not necessarily demonstrate that these capabilities can be translated into sustained productivity gains at commercial scale. This distinction between technological capability and industrial effectiveness is central to a critical assessment of the field: knowing what is happening inside the reactor is a necessary condition for precision management, but it is not sufficient unless sensing, prediction, and intervention can operate as an integrated feedback system under production-relevant conditions. *Precision microalgae* should therefore not be interpreted as a ready-made technological solution, but as a research framework whose industrial value will ultimately depend on whether improved observability and biological control can be converted into reproducible gains in productivity, robustness, and resource-use efficiency at scale.

## 6. Translational Bottlenecks: From Laboratory Success to Industrial Reality

Despite substantial advances in sensing, computational modeling, artificial intelligence, and bioengineering, microalgal production remains restricted to a relatively small number of commercial applications. The central challenge is no longer demonstrating that technology works under controlled conditions but ensuring that it remains effective when transferred to biologically variable industrial environments.

Demonstrating technical feasibility is not equivalent to demonstrating industrial viability. The recent history of microalgal biotechnology is marked by this discrepancy: strategies that perform exceptionally well under laboratory conditions frequently lose effectiveness when confronted with environmental variability, economic constraints, and regulatory requirements characteristic of real-world production systems [4].

After more than seven decades of research, only a limited number of microalgal products have reached commercial maturity, mainly concentrated on high-value applications such as phycocyanin, astaxanthin, and omega-3 fatty acids [4]. This persistent gap between laboratory success and industrial implementation does not arise from a lack of scientific knowledge. On the contrary, the literature provides an expanding portfolio of tools for monitoring, modeling, and engineering microalgal systems. The difficulty lies in transferring these advances to environments where variability is not minimized but rather constitutes a defining characteristic of the production process.

The first translational bottleneck is reproducibility. In laboratory environments, irradiance, temperature, medium composition, reactor geometry, and mixing conditions are maintained within narrow operational windows. Industrial cultivation systems, particularly outdoor facilities, are exposed to continuous fluctuations driven by weather conditions, seasonality, and operational heterogeneity [4,21]. As a consequence, the same strain may exhibit substantially different performances without any change in its genetic composition. What changes is the environment in which the strain operates. This observation carries important implications: many improvements reported in the literature reflect properties of the experimental system rather than intrinsic properties of the strain itself.

Quantitative evidence further illustrates the magnitude and diversity of this translational gap. As summarized in Table 5, changes in production scale are associated with different degrees of performance loss depending on the cultivation system, environmental exposure, reactor configuration, and duration of operation. Importantly, these comparisons also indicate that scale-up is rarely an isolated variable, since increasing production scale is frequently accompanied by changes in environmental control, reactor geometry, cultivation strategy, and downstream processing.

Taken together, these quantitative comparisons show that the laboratory-to-industrial yield gap cannot be attributed to cultivation volume alone. The nature of the comparison differs among studies: while the *Scenedesmus* case represents direct geometric scale-up, *Haematococcus pluvialis* illustrates the transition from controlled indoor to variable outdoor conditions, and the *Nannochloropsis* comparison isolates the influence of reactor configuration under the same climatic conditions. At the commercial scale, *Arthrospira platensis* further demonstrates that seasonal variability and downstream harvesting losses can substantially reduce realized productivity during long-term operation. Conversely, the *Chlorella vulgaris* case shows that such bottlenecks can be partially overcome by redesigning the scale-up pathway itself rather than simply increasing cultivation volume. Therefore, “scale-up” should be interpreted as a multidimensional transition involving changes in reactor geometry, environmental variability, cultivation strategy, operational duration, and downstream processing. Performance losses generically attributed to scale may thus reflect the cumulative loss of physical and operational control that accompanies the transition toward production-relevant environments.

This challenge becomes particularly evident in cellular bioengineering. Most engineering strategies are designed to enhance specific characteristics, such as growth, lipid accumulation, carbon assimilation, or stress tolerance. However, these modifications are generally validated under highly controlled conditions. When transferred to heterogeneous industrial environments, their advantages frequently diminish or disappear [131]. Not because the engineering approach failed, but because the environmental assumptions that supported the desired phenotype no longer exist.

The second bottleneck is long-term biological stability. Genetically engineered strains are often evaluated over periods of days or weeks, whereas industrial cultivation requires stable performance over months or repeated production cycles. Under these conditions, evolutionary processes continue to operate. Cells that reduce the metabolic burden associated with maintaining transgenes or synthetic circuits may gain a competitive advantage over those that preserve these functions [132]. Over time, this selection pressure can progressively alter population composition and compromise the engineered phenotype, particularly in continuous and semi-continuous cultivation systems [131,132].

The third bottleneck is regulatory and social acceptance. Industrial adoption depends not only on technical performance but also on biosafety validation, environmental risk assessment, genetic stability evaluation, and compliance with regulatory frameworks that differ among countries and markets [133]. In food, nutraceutical, and cosmetic applications, these requirements become even more stringent. Furthermore, public perception of genetically modified organisms remains heterogeneous and is often associated with concerns regarding environmental release and biosafety, especially in open cultivation systems [134]. Consequently, the pace of technological innovation often outpaces the evolution of regulatory frameworks and societal acceptance.

Perhaps the most important bottleneck identified throughout this review, however, is neither exclusively technological, biological, nor regulatory. It is conceptual. The quantitative performance gaps summarized above reinforce a broader pattern already evident across macro and microspatial scales: many optimization strategies continue to rely on the implicit assumption that one variable can be improved without significantly affecting others. The evidence discussed throughout this article suggests the opposite. In microalgal systems, virtually every relevant optimization generates trade-offs in another dimension of performance.

Increasing light intensity promotes photosynthesis, but it accelerates the accumulation of dissolved oxygen and increases the incidence of photoinhibition. Intensifying mixing reduces mass gradients but increases mechanical stress and energy consumption. Inducing nutritional stress increases lipid content but reduces biomass productivity. Genetically modifying metabolic pathways to direct carbon toward specific products often reduces growth or physiological robustness. The problem is not that these strategies are wrong. The problem is that they operate within a system whose nature is intrinsically integrated.

Viewed collectively, these examples reveal that industrial microalgal cultivation is governed not by isolated optimization targets, but by a network of interconnected trade-offs. As a result, gains achieved in one aspect of performance often come at the expense of another. Box 3 summarizes some of the most common trade-offs that emerge when biological, engineering, and operational objectives are optimized simultaneously.

Box 3 **Structural trade-offs in microalgal bioengineering: when optimizing one function compromises another.**

**Figure 6:**
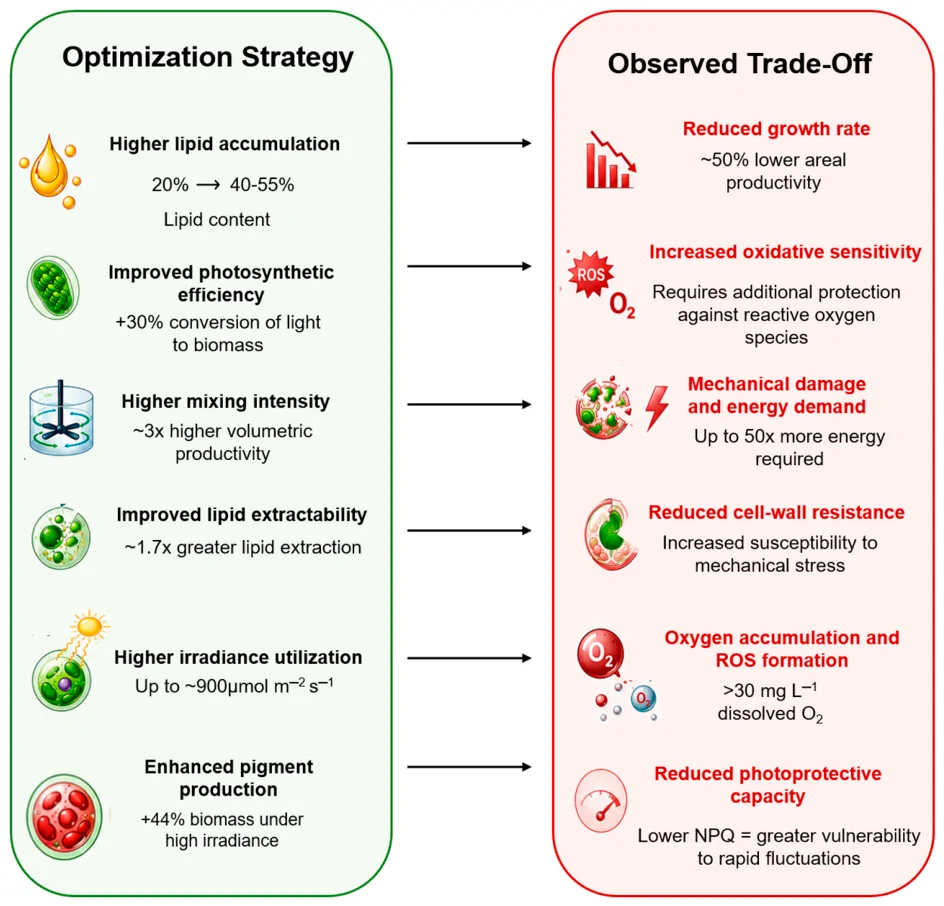
Representative optimization strategies and their associated trade-offs in microalgal cultivation and bioengineering. Original conceptual illustration created by the authors based on quantitative evidence and optimization–trade-off relationships reported in Refs. [48,62,85,94,107].

The translational bottlenecks discussed throughout this section reveal a recurring pattern in microalgal biotechnology: advances that address a specific limitation often generate new constraints at other stages of the process. In other words, industrial performance is rarely governed by a single isolated factor, but rather by a network of technical, biological, operational, and economic trade-offs. Figure 6 conceptually synthesizes representative optimization strategies and their associated trade-offs, illustrating how improvements in productivity, robustness, or efficiency are frequently accompanied by biological, operational, or energetic constraints elsewhere in the system.

The trade-offs illustrated in Figure 6 should not be interpreted as failures of current technologies. Rather, they represent emergent properties of complex biological systems operating under unavoidable energetic, metabolic, and physical constraints. As highlighted by the optimization–trade-off relationships, improving one biological or engineering function frequently reallocates resources or introduces new limitations elsewhere in the system. Consequently, the industrial challenge is not to eliminate these trade-offs, but to understand, anticipate, and manage them through integrated cultivation, reactor engineering, and adaptive bioengineering strategies.

Recognizing these trade-offs shifts the focus from optimization to management. The relevant question is no longer which modification maximizes productivity under ideal conditions, but how productive performance can be maintained under the biological and environmental variability that characterizes industrial cultivation. Addressing this challenge requires moving beyond isolated technological advances toward integrated and adaptive production systems.

## 7. Future Directions: Toward an Operational Precision Microalgae Framework

The challenge identified throughout this review naturally leads to a broader question: what type of production system can remain functional, productive, and economically viable when exposed to the environmental variability that defines industrial-scale cultivation?

A practical example helps illustrate this challenge. In an outdoor tubular photobioreactor cultivating a lipid-producing *Nannochloropsis* strain, dissolved oxygen concentrations may exceed 30 mg L^─1^ during periods of peak irradiance. Under such conditions, photosynthetic activity initially increases, but the accumulation of reactive oxygen species (ROS) can trigger antioxidant responses, alter carbon partitioning, and ultimately reduce lipid productivity and biomass stability [48,49]. In conventional cultivation systems, these physiological changes may remain undetected until productivity losses become evident.

Within a *precision microalgae* framework, the same event would trigger a fundamentally different sequence of responses. Sensors monitoring dissolved oxygen, fluorescence, and culture performance would detect physiological changes in real time [135] A continuously updated digital twin integrating hydrodynamic, physiological, and metabolic information could interpret these signals as indicators of impending oxidative stress rather than normal operational fluctuations [25]. Process-control systems could then automatically adjust aeration rates, circulation intensity, or light exposure before productivity losses become established. If combined with programmable cellular platforms capable of responding to oxidative signals, protective physiological responses could be activated before irreversible damage occurs [109,132].

This scenario does not describe a fully established technology. Rather, it illustrates the operational logic underlying the *precision microalgae* paradigm. In this framework, adaptive cultivation systems constitute the intervention layer, dynamically adjusting operating conditions according to the physiological state of the culture. Integrated digital platforms, combining distributed sensors, digital twins, and machine-learning algorithms, provide the interpretative layer responsible for distinguishing normal variability from early signs of biological instability. Programmable cellular platforms add a third layer by enabling targeted physiological responses to specific environmental stimuli.

Supporting these components is the integration of cellular engineering, process engineering, sensing technologies, and artificial intelligence from the earliest stages of technology development. Instead of following the traditional linear pathway in which biological optimization precedes engineering implementation, this approach promotes iterative and multiscale design, where industrial performance is considered from the outset of biological development [107,108].

From this perspective, *precision microalgae* is not a technology to be installed but a design philosophy that treats biological and engineering systems as a single object of optimization. Its relevance does not lie in eliminating the trade-offs discussed throughout this review, but in making them observable, predictable, and operationally manageable. Whether this vision can be fully translated into industrial reality remains an open question. Achieving this goal will require advances in sensing, computational modeling, synthetic biology, and adaptive process control to evolve beyond isolated technological solutions and become components of a unified operational platform. *Precision microalgae* should therefore not be viewed as the end of the trade-offs that define industrial cultivation, but as a framework for understanding, anticipating, and managing them more effectively. In this sense, the production system capable of remaining functional under industrial variability is not necessarily the one that eliminates fluctuations, but the one that continuously detects, interprets, and adapts to them*. Precision microalgae* represent a conceptual framework for enabling this transition.

## 8. Conclusions

The macrospatial bottlenecks associated with light distribution, gas transfer, thermal gradients, and hydrodynamics, as well as the microspatial bottlenecks related to cell-cycle regulation, carbon assimilation, oxidative stress, and physiological heterogeneity, do not constitute independent problems. Rather, they represent different manifestations of the same underlying phenomenon: the difficulty of efficiently connecting the physical dynamics of cultivation systems with the biological dynamics of the cells. The primary barrier to the industrial scale-up of microalgae lies neither exclusively in the limitations of photobioreactors nor in the metabolic capabilities of individual strains, but in the inability of current systems to adequately understand and respond to the variability that emerges from the interaction between the two.

The advances achieved over the past decades demonstrate that significant progress has been made across multiple fronts. Nevertheless, these approaches remain largely implemented in a fragmented manner. As a result, gains achieved in one dimension are often constrained by limitations that emerge in another, hindering the consistent translation of laboratory-scale achievements to industrial production. In this context, an integrative paradigm such as *precision microalgae* may help guide the next generation of microalgal biotechnology. Rather than treating physiological, environmental, and operational heterogeneity as a source of uncertainty to be eliminated, this approach recognizes it as a source of information capable of guiding cultivation decisions, control strategies, and strain-development efforts. The concept is not based on specific technology, but on the integration of real-time monitoring, predictive modeling, artificial intelligence, bioengineering, and adaptive control, enabling the inherent variability of biological systems to be incorporated into the decision-making process.

At present, the most important contribution is not to propose a definitive technological solution, but to offer a new way of interpreting the scale-up challenge. From this perspective, industrial success no longer depends on the search for an ideal strain or a perfectly homogeneous cultivation system, but on the ability to understand, predict, and manage the variability that characterizes complex biological systems. Scalability, therefore, does not emerge from the elimination of heterogeneity, but from the capacity to transform it into operational information. It is within this conceptual shift that the principal contribution and transformative potential of the paradigm proposed in this review reside.

## Figures and Tables

**Table 1:** Quantitative effects of major macrospatial bottlenecks on microalgal cultivation and process performance at production-relevant scales.

Macrospatial Bottleneck	Species/Cultivation System and Scale	Operational/Environmental Parameter	Quantitative Effect on Production Performance	Main Trade-Off/Implication
Reactor geometry-light distribution	*Nannochloropsis* sp.; modeled commercial-scale outdoor flat-panel PBR vs. open raceway [32]. *Chlorella vulgaris*; 128–684-mL laboratory V-shaped and flat PBRs [34]	Reactor configuration and light utilization	Modeled areal productivity was 10.4–35.6 g m^−2^ d^−1^ in flat-panel PBRs versus 5.5–19.7 g m^−2^ d^−1^ in raceways [32]. Experimentally, a V-shaped PBR increased productivity from 20.7 to 52.0 g m^−2^ d^−1^ and photosynthetic efficiency from 1.40% to 3.52% [34]	Geometry can improve light utilization substantially, but experimental gains obtained in small controlled systems still require outdoor and production-scale validation.
Hydrodynamics–geometry-dependent dead zones	Pilot-scale high-depth raceway pond (4.5 m^3^); CFD model validated by PIV [35].	Local circulation velocity, flow deflectors, and reactor length-to-width ratio.	Regions with velocity < 0.1 m s^−1^ were classified as dead/stagnant zones; flow deflectors reduced dead zones by 28%, and pressure drop across 180° bends by 49%; optimal L/W ≈ 10 [35].	Geometry determines the effectively mixed culture volume; reducing stagnant regions may improve mass renewal while lowering hydraulic losses.
Seasonal variability	*Limnospira platensis*; industrial raceways of 1000 and 4000 m^2^ [38]. *Nannochloropsis oculata*; 2.5-m^3^ outdoor pilot raceway operated for 16 months [39].	Seasonal solar radiation and temperature	*L. platensis* showed annual productivity of 5.1–5.6 g m^−2^ d^−1^ and a >60% winter decline relative to peak yields of 7.5–7.6 g m^−2^ d^−1^ [38]. *N. oculata* productivity increased from 0.72–1.27 g m^−2^ d^−1^ in winter to 3.62 g m^−2^ d^−1^ in summer [39]	Peak-season productivity can substantially overestimate sustained annual performance; seasonal responses also depend on strain, location, and contamination.
Thermal stress	*Picochlorum* sp. BPE23; 1.8-L laboratory flat-panel PBR under simulated tropical diel cycles [44]. *Dunaliella salina*; modeled full-scale outdoor vertical PBRs [66]	Exposure above the critical temperature threshold (~43 °C)	For *Picochlorum*, daily dilution rate decreased from 1.22 ± 0.06 d^−1^ at a 40 °C peak to 0.61 ± 0.03 and 0.29 ± 0.02 d^−1^ at 45 and 47.5 °C, respectively [44]. Modeling predicted culture collapse on 76 and 131 d yr^−1^ and annual productivity losses of approximately 35% and 40% [66].	Thermotolerant strains may remain productive at 40 °C, but further warming sharply reduces performance; tolerance is strain-specific and controlled results do not ensure annual outdoor robustness.
Gas transfer- O_2_ accumulation	*Chlorella vulgaris* laboratory *PBR* [48]. *Monoraphidium minutum* and *Scenedesmus obliquus*; laboratory oxygen-response assays and 100-L outdoor raceways [49].	Dissolved O_2_ accumulation and removal by air sparging.	Approximately 31 mg O_2_ L^−1^ reduced *C. vulgaris* productivity by ~30% [48]. At 31 mg O_2_ L^−1^, photosynthetic rates fell by 36.6% in *M. minutum* and 26.1% in *S. obliquus*. Outdoor air sparging lowered DO by 10–20 mg L^−1^ and increased *S. obliquus* productivity by 36%, from 13.9 ± 0.4 to 18.9 ± 1.6 g m^−2^ d^−1^ [49]	O_2_ removal can improve productivity, but effectiveness depends on strain and season and must be balanced against energy aeration requirements.
Nutrient stoichiometry–C/N imbalance	Mixed microalgal communities; outdoor pilot high-rate algal ponds treating blended wastewater [52].	Influent C/N ratios of 9.65, 25.31, and 52.71	Biomass productivity decreased from 2.35 to 1.96 and 1.63 g VSS m^−2^ d^−1^ whereas carbohydrate content increased to 18.44% and 21.09% at the two higher C/N ratios [52]	Stoichiometry affects biomass formation and biochemical partitioning; the preferred C/N depends on whether biomass or storage products are targeted.
Hydrodynamics-mixing	*Nannochloropsis oceanica*; pilot raceways ~28 m^2^ [59].	Circulation velocity and daily mixing duration.	Increasing velocity from 0.15 to 0.30 m s^−1^ raised areal productivity by 1.7 g m^−2^ d^−1^ and lipid content by ~33% but doubled specific power consumption [59].	More intensive mixing improves transport and productivity but increases energy demand; reduced mixing may promote gradients, settling, and dead zones.
Microbial contamination–bacterial predation	*Chlorella* sp.; outdoor raceways from 1000 L validation units to 130,000 L commercial ponds [60].	*Vampirovibrio chlorellavorus* infection and acidification-based control treatment	Bacterial attachment increased from ~20% to 100% within three days, followed by collapse within 2–5 days; treatment increased mean culture longevity from 7.0 ± 2.7 to 12.7 ± 2.7 days [60]	Microscale predator–cell interactions can rapidly cause the loss of entire commercial cultures; detection and control must be integrated into large-scale operation.
Microbial contamination–fungal parasitism	*Graesiella* sp. WBG-1; outdoor raceways up to 1000 m^2^ and 200,000 L [61].	Fungal parasite treatment with 10 mg L^−1^ SDBS	SDBS prevented fungal infection at pilot and industrial scales without reducing biomass production or lipid content; cultivated biomass maintained >30% lipid content [61]	Control may preserve culture stability, but mitigation remains species- and contaminant-specific.
Shear stress-mechanical damage	*Limnospira platensis*; 2.6 m^3^ pilot tubular PBR versus raceway [62].	Centrifugal pumping/high shear	Tubular PBR productivity was 0.154 ± 0.030 versus 0.049 ± 0.001 g L^−1^ d^−1^ in raceway, despite ~90% filament fragmentation within 24 h [62]	Higher volumetric productivity can mask severe morphological damage and substantially greater energy demand.
Process integration–net energy balance	Continuous pilot microalgal–bacterial PBR treating wastewater; biomass-to-biodiesel pathway [63].	Expansion of the system boundary from biomass production to lipid extraction and biodiesel conversion.	Net energy ratio decreased from 4.95–8.38 for biomass production to 0.14–0.23 for the complete biodiesel pathway; downstream operations accounted for ~88–90% of cumulative energy demand [63]	Positive upstream performance does not ensure positive process-level energy recovery; viability depends on downstream operations and system boundaries.

**Table 2:** Quantitative outcomes and translational relevance of representative cellular bioengineering strategies in microalgae.

Engineering Target	Strategy/Microalga	Quantitative Outcome and Validation Context	Translational Significance
Carbon assimilation	RuBisCO activase-like protein overexpression in *N. oceanica* [92]. RuBisCO overexpression in *Synechocystis* sp. PCC 6803 [93]	In *N. oceanica*, growth increased by ~32%, biomass by ~46%, and lipid productivity by ~41% under atmospheric CO_2_, but the growth advantage declined to ~3% at 5% CO_2_ [92]. In *Synechocystis*, RuBisCO overexpression increased growth rate from 0.175 ± 0.010 to 0.187 ± 0.004 OD730 d^−1^ under 20 mM NaHCO_3_ [93]	Laboratory cultivation. Both responses depended strongly on inorganic-carbon availability, and neither strategy was validated outdoors.
Inorganic carbon acquisition	Heterologous MlCA overexpression in *Chlorella vulgaris* and *C. sorokiniana* [84]. CrLCIA bicarbonate-transporter expression in *N. salina* [94]	MlCA increased carbonic-anhydrase activity by 23–31%, reaching 156.6–181.2 WAU mL^−1^ and increased biomass 1.6–2.0-fold after 7 d under 1% CO_2_ [84]. Growth increased by ~30%, and biomass approximately doubled under low-CO_2_ conditions [94].	Laboratory cultivation; MlCA was evaluated as a two-layer photoreactor. Benefits depended on CO_2_/HCO_3_^−^ availability, without outdoor validation.
Photoprotective regulation	Reduced NPQ in *C. vulgaris* [95]. controlled LHCSR3 expression in *Chlamydomonas reinhardtii* [96]	Reduced NPQ increased biomass by up to 44% under high irradiance [95]. Controlled LHCSR3 expression increased maximal daily productivity by ~35%, final biomass by ~34%, and photosynthetic efficiency from 3.4% to 4.4–4.7% [96].	Laboratory cultivation under controlled high irradiance. Productivity gains required sufficient residual photoprotection, and outdoor stability was not evaluated.
Light-harvesting efficiency	Reduced photosynthetic antenna in TAM-2 *C. sorokiniana* [85]. reduced-antenna and oxidative-stress-resistant *C. vulgaris* mutants [97]	Biomass productivity increased from 290 to 380 mg L^−1^ d^−1^ (~32%) in a 1-L laboratory PBR, with a productivity advantage also observed in outdoor PBR cultivation [85]. In *C. vulgaris*, reduced antenna size increased biomass productivity by ~28%, while its combination with ^1^O_2_ resistance increased productivity from 430 to ~700 mg L^−1^ d^−1^ [97]	TAM-2 was validated in 1-L indoor and 7-L outdoor PBRs, where its advantage over WT was maintained under natural light and temperature fluctuations. The *C. vulgaris* mutants were validated in 1-L laboratory PBRs.
Carbon partitioning toward lipids	ZnCys transcriptional-regulator attenuation in *N. gaditana* [86]. heterologous AtL1L expression in *Phaeodactylum tricornutum* [98]	ZnCys attenuation increased areal lipid productivity from ~2.5 to ~5.0 g m^−2^ d^−1^ and carbon partitioning to lipids from ~20% to 40–55% [86]. AtL1L increased lipid content from 20.9% to 29.8–33.9% of dry weight while decreasing carbohydrates from 23.1% to 13.3–16.5%; total fatty acids increased by 48–68% [98].	Laboratory cultivation. AtL1L redirected carbon toward lipids without significantly compromising biomass yield or Fv/Fm, but neither strategy was validated outdoors.
Lipid-biosynthesis pathway	Combined GPAT/DGAT overexpression in *P. tricornutum.* NeoDGAT2 overexpression in *Neochloris oleoabundans* [99]	Combined GPAT/DGAT overexpression increased lipid accumulation by ~2.6-fold [82]. NeoDGAT2 increased TAG content by 1.8–3.2-fold and TAG productivity by 1.6–4.3-fold, reaching 8.9 ± 1.3 mg L^−1^ d^−1^ [99].	Laboratory cultivation, including nitrogen-starvation conditions. Strong lipid phenotypes were demonstrated, but environmental robustness and outdoor productivity were not evaluated.
Metabolic redirection	CRISPRi-mediated PEPC repression in *C. reinhardtii.* DNA-free CRISPR–Cas9 knockout of AGP in *Tetraselmis* sp. [100]	PEPC repression increased lipid productivity by 94.2% [83]. AGP knockout reduced starch to ~0.9% of dry weight and increased fatty-acid productivity from 11.06 ± 1.56 to 24.86 ± 2.41–29.68 ± 7.70 mg L^−1^ d^−1^ [100].	Laboratory cultivation. Both strategies redirected carbon from competing pathways toward lipids, but their performance under outdoor fluctuations remains unknown.

**Table 3:** Quantitative evidence and validation scale of representative precision strategies in microalgal cultivation.

Precision Strategy	Quantitative Evidence	Validation Scale
Biomass monitoring and culture-state estimation	Turbidity-based monitoring achieved R^2^ = 0.943–0.986 for optical density and R^2^ = 0.954–0.975 for dry weight [14]. XGBoost-based growth prediction reached training R^2^ = 0.9997 [24]. Under less controlled conditions, Random Forest achieved only R^2^ = 0.66 (dry weight) [110] An ML-based harvest decision-support framework matched human-guided productivity while reducing variability in commercial operation [111]	*Limnospira fusiformis*: 0.9-L indoor cultivation, with automated biomass regulation also evaluated in a 4.5-L outdoor flat-panel PBR [14]. *Chlorella* sp.: indoor tubular PBR [24]. *Scenedesmus dimorphus*: laboratory PBR [110]. *Nannochloropsis* sp.: 12 commercial tubular PBRs, 3 years of farm data [111].
Adaptive operation and dynamic setpoints	Changing the turbidostat biomass threshold from 1.0 to 0.8 g L^−1^ increased harvested areal productivity from 9.52 to 23.20 g m^−2^ d^−1^ [23]. Model-guided nutrient supply reduced glucose and nitrate consumption by 75% and 23%, respectively, while closed-loop GMPC reduced glucose-setpoint control error by approximately 80% relative to PID [104]. In pH-controlled cultures, NH_3_ volatilization decreased from 29.6–71.0% to <5% [105]. FIPO increased simulated biomass production by 21.3% vs. batch and 7.4% vs. fixed dilution; long-term gains were 13.3–17.8% [112]. EMPC produced > 33% higher simulated profit than conventional operation over 14 days [113].	*Scenedesmus almeriensis*: 12,000-L/80-m^2^ outdoor raceway, operated for 14 days [2]. *Chlorella vulgaris*: 2-L laboratory bioreactors [104]. Mixed microalgal culture: 0.5-L laboratory flask cultures [105]. In silico; growth model validated with *C. sorokiniana* in two 800-L indoor raceways simulating outdoor light (NSE = 0.94–0.99) [112]. In silico; 80-m^2^ semi-industrial raceway model driven by real seasonal data; no closed-loop field validation [113].
Sensor- and image-based diagnostics	Automated image analysis classified *Scenedesmus coenobia* with 98.63% accuracy using SVM and 97.32% using ANN [114]. A low-cost monitoring system showed relative pH measurement errors of approximately 0.2–0.3%, DO deviations of 0.13–0.84%, and a mean microalgal-density estimation error of 8.6 ± 1.8% relative to direct microscopic counts [115]. EfficientNet and ResNet predicted algal concentration with R^2^ = 0.94–0.99 in an independent validation dataset [116].	*Scenedesmus* spp.: laboratory/off-line microscopic image analysis [114]. *Chlorella vulgaris*: continuous closed cultivation system; pH and DO were validated against reference measurements, while density estimates were compared with direct microscopic counts across 25 solutions ranging from 10.5 to 42.3 million cells mL^−1^ [115]. Dried *Rhodophyta* and *Spirulina* powder under controlled illumination; no live-culture validation [116].
Adaptive light control	Lumostatic cultivation of *Chlorella* sp. reached 5.78 g L^−1^ maximum biomass and 1.29 g L^−1^ d^−1^ productivity, corresponding to +25.7% maximum biomass and +74.3% productivity compared with the best constant-light condition [88]. A light-driven dilution strategy (OptiLum) increased productivity by 86–97% [117]. Biomass-responsive lighting combined with CFD-optimized mixing increased biomass productivity by 20%, carbon fixation by 43%, and phycocyanin content by 14.4% [118].	*Chlorella* sp.: 1.5-L laboratory draft-tube PBR; irradiance was adaptively increased according to culture development, reaching a maximum supplied irradiance of 590 µmol m^−2^ s^−1^ [88]. OptiLum: outdoor operation within the DISCOVR consortium [117]. *Limnospira fusiformis*: 71.4 L indoor raceway; integrated light–mixing strategy, without outdoor validation [118].
Predictive and model-based cultivation management	An analytical formula estimated maximum volumetric biomass productivity across photobioreactors of different designs with deviations of approximately ±15% from the experimental values [107]. A thermal–biological model reproduced outdoor observations within approximately ±8.4% over 148 days of multiseason operation [119]. Four models trained with laboratory data showed reduced predictive performance when transferred to ten outdoor pilot-PBR cultivations; the best-performing model achieved RMSE = 0.296 [120].	*Arthrospira platensis*; experimental validation across photobioreactors of different designs and a wide range of cultivation volumes and incident light fluxes [107]. Thermal/biological model: ATP3 consortium data, multiple species/sites/seasons [119]. *Nannochloropsis* sp.: 10 cultivations in an outdoor pilot closed PBR [120].
Genetic engineering for performance and robustness	Attenuation of the ZnCys transcriptional regulator in *Nannochloropsis gaditana* increased areal lipid productivity from ~2.5 to ~5.0 g m^−2^ d^−1^ (~2-fold; up to +103%), with only a ~5–15% decrease in total organic carbon productivity [86]. Overexpression of NsbHLH2 achieved FAME productivity of 83.6 mg L^−1^ d^−1^ under continuous cultivation [121]. Overexpression MlCA increased lipid production 2.2-fold, reaching 1.1 g L^−1^ [84].	*N. gaditana*: 550-mL semicontinuous laboratory culture under simulated diel irradiance [86]. *N. salina*: laboratory continuous culture [121]. *C. sorokiniana* and *C. vulgaris*: laboratory two-layer photoreactor [84]; no outdoor validation

The quantitative values summarized in this table originate from independent studies involving different organisms, cultivation systems, operating conditions, experimental durations, validation scales, performance indicators, and reference baselines. They are presented as representative evidence of the reported effects and validation levels and should not be interpreted as directly comparable values, a ranking of strategies, or a pooled quantitative analysis.

**Table 5:** Quantitative evidence of translational performance differences across laboratory, pilot, outdoor, and industrial microalgal cultivation.

Microalgae	Target Product/System	Small-Scale	Larger-Scale/Production-Relevant Performance	Transition	Performance Change During Scale-Up
*Scenedesmus* spp.	Biomass; flat-plate gas-lift PBR	1.8-L laboratory PBR	Geometrically equivalent 300-L pilot PBR	Laboratory → pilot scale	Volumetric and areal productivity decreased by up to 11% and approximately 7.5%, respectively [122]
*Chlorella vulgaris*	Biomass; heterotrophic inoculum strategy	5-L heterotrophic fermenter: 27.3 ± 6.8 g L^−1^ d^−1^	0.2 and 5 m^3^ fermenters: 27.54 ± 5.07 and 31.86 ± 2.87 g L^−1^ d^−1^; inocula subsequently transferred to 100-m^3^ outdoor PBRs	5 L/0.2 m^3^/5 m^3^/100 m^3^ outdoor PBRs	Fermentation productivity was maintained, while scale-up time and occupied area decreased fivefold and twelvefold, respectively [123]
*Nannochloropsis gaditana Lubián* CCMP 527	Biomass; continuous cultivation	Indoor PBR: 0.49 g L^−1^ d^−1^	Three 340-L outdoor tubular PBRs: 0.59 g L^−1^ d^−1^	Laboratory cultivation → outdoor pilot	Maximum volumetric productivity increased by approximately 20%, despite changes in geometry and environmental conditions [124,125]
*Haematococcus pluvialis*	Astaxanthin; continuous one-step cultivation	Laboratory cultivation: 20.8 ± 2.8 mg L^−1^ d^−1^	Outdoor tubular PBR: 8 mg L^−1^ d^−1^	Controlled laboratory → outdoor	Astaxanthin productivity decreased by approximately 62% [126,127]
*Nannochloropsis oculata* CCMP 525 and *N. salina* CCMP 1776	Biomass and biooil; industrial outdoor PBR	Peak biomass productivity: 0.37 g L^−1^ d^−1^; peak bio-oil production: 21.1–36.3 m^3^ ha^−1^ yr^−1^	Over 2.5 years: 0.15–0.16 g L^−1^ d^−1^; average bio-oil production: 7.04–13.1 m^3^ ha^−1^ yr^−1^	Peak performance → sustained industrial operation	Sustained biomass and bio-oil performance were approximately 57–59% and 64–67% below peak values, respectively [128]
*Schizochytrium* sp. CCTCC M209059	DHA; fed-batch fermentation at constant kLa	100-mL flasks: 101.8 mg L^−1^ h^−1^	1500 and 7000 L: 101.1 and 136.9 mg L^−1^ h^−1^	100 mL → 10 and 50 L → 1500 and 7000 L	HA productivity was maintained at 1500 L and increased by approximately 34% at 7000 L when kLa was preserved at 88.9 h^−1^ [129]
*Limnospira platensis*	Biomass and phycocyanin; industrial raceways	Summer: 7.3–7.6 g m^−2^ d^−1^; phycocyanin content of 15.2%	One-year operation in 1000- and 4000-m^2^ raceways: annual average of 5.1–5.6 g m^−2^ d^−1^; winter productivity of 1.7–2.8 g m^−2^ d^−1^ and phycocyanin content of 10.4%	Favorable season → year-round industrial operation	Winter biomass productivity was approximately 63–77% lower; phycocyanin content decreased by approximately 32%, and its productivity was more than threefold lower [38,130]

## Data Availability

The authors declare that all data supporting the conclusions of this study are available within the article itself, through the duly cited sources. No original datasets were generated in this study. The literature analyzed was obtained from the database, following the search strategy described in the manuscript. The studies used to support the analyses and discussions are duly cited throughout the article.
